# Cytotype Affects the Capability of the Whitefly Bemisia tabaci MED Species To Feed and Oviposit on an Unfavorable Host Plant

**DOI:** 10.1128/mBio.00730-21

**Published:** 2021-11-16

**Authors:** Sylvain Benhamou, Isabelle Rahioui, Hélène Henri, Hubert Charles, Pedro Da Silva, Abdelaziz Heddi, Fabrice Vavre, Emmanuel Desouhant, Federica Calevro, Laurence Mouton

**Affiliations:** a Université de Lyon, Université Lyon 1, CNRS, VetAgro Sup, Laboratoire de Biométrie et Biologie Evolutive, UMR 5558, Villeurbanne, France; b Univ Lyon, INRAE, INSA Lyon, BF2I, UMR 203, Villeurbanne, France; Johannes Gutenberg University of Mainz; University of Hawaii at Manoa

**Keywords:** cytotype, symbionts, plant utilization, *Bemisia tabaci*

## Abstract

The acquisition of nutritional obligate primary endosymbionts (P-symbionts) allowed phloemo-phageous insects to feed on plant sap and thus colonize novel ecological niches. P-symbionts often coexist with facultative secondary endosymbionts (S-symbionts), which may also influence their hosts’ niche utilization ability. The whitefly Bemisia tabaci is a highly diversified species complex harboring, in addition to the P-symbiont “*Candidatus* Portiera aleyrodidarum,” seven S-symbionts whose roles remain poorly understood. Here, we compare the phenotypic and metabolic responses of three B. tabaci lines differing in their S-symbiont community, reared on three different host plants, hibiscus, tobacco, or lantana, and address whether and how S-symbionts influence insect capacity to feed and produce offspring on those plants. We first show that hibiscus, tobacco, and lantana differ in their free amino acid composition. Insects’ performance, as well as free amino acid profile and symbiotic load, were shown to be plant dependent, suggesting a critical role for the plant nutritional properties. Insect fecundity was significantly lower on lantana, indicating that it is the least favorable plant. Remarkably, insects reared on this plant show a specific amino acid profile and a higher symbiont density compared to the two other plants. In addition, this plant was the only one for which fecundity differences were observed between lines. Using genetically homogeneous hybrids, we demonstrate that cytotype (mitochondria and symbionts), and not genotype, is a major determinant of females’ fecundity and amino acid profile on lantana. As cytotypes differ in their S-symbiont community, we propose that these symbionts may mediate their hosts’ suitable plant range.

## INTRODUCTION

Microbial symbionts have been associated in numerous phytophagous insects with adaptive changes that profoundly influence their interactions with host plants. For instance, symbionts can supplement their hosts with essential and otherwise limiting nutrients, detoxify plant defense compounds, or break down plant polymers (1, 2). In sap-feeding hemipteran insects, the acquisition of nutritional “primary” obligate bacterial endosymbionts (P-symbionts) is considered a pivotal evolutionary event that allowed them to thrive on plant sap, a diet where amino acids and vitamins essential to their growth are limited (3, 4). P-symbionts are strictly maternally inherited and are intracellular, housed in the cytoplasm of specialized host cells, the bacteriocytes, that constitute symbiosis-dedicated organs, the bacteriomes (5), localized in the insect abdomen (6–8).

In addition to P-symbionts, hemipterans often carry “secondary” facultative endosymbionts (S-symbionts) that are not essential for their hosts’ survival. S-symbiont-mediated phenotypes are diverse. Some S-symbionts are reproductive parasites biasing sex ratios in favor of daughters or inducing incompatibility in uninfected zygotes (for a review, see reference 9); others are mutualists and confer benefits to their hosts (10, 11), such as protection against natural enemies (12) or thermal tolerance (13). S-symbionts can be either localized inside or outside the bacteriocytes, and, although their transmission is mainly vertical, they can also be transmitted horizontally (14), for instance, through the host plant when individuals share the same feeding sites (15, 16).

Given their labile nature, S-symbionts are suggested to form a “horizontal gene pool,” and their acquisition can confer beneficial traits and contribute to the host adaptation to novel ecological niches (17, 18). Indeed, P-symbionts genomes are particularly prone to erosion and thus to a decay of their metabolic functions (19–22); S-symbionts may complement or replace parts of the degenerated functions that P-symbionts can no longer fulfill. As an example, “*Candidatus* Serratia symbiotica” S-symbiont has become a coprimary symbiont in the aphid Cinara cedri and contributes to the symbiotic metabolism by producing tryptophan, which cannot be synthetized by the reduced genome of the P-symbiont, Buchnera aphidicola (23). Aphid S-symbionts have also been suggested to contribute to host plant adaptation, leading to specialized host-adapted races, but this hypothesis remains controversial. Several studies failed to support an S-symbiont-mediated plant utilization (24–26), but other works found a significant relationship between the adaptation to a given host plant and the presence of specific S-symbionts within (27–29) and across (17, 30) aphid species. As an example, in the pea aphid Acyrthosiphon pisum, the S-symbiont “*Candidatus* Regiella insecticola” has been associated with the use of the white clover Trifolium repens (28). Similarly, the S-symbiont *Arsenophonus* has been associated with the specialization on the black locust Robinia pseudoacacia in the aphid Aphis craccivora (31).

Bemisia tabaci (Hemiptera: Aleyrodidae) is a highly diversified complex of morphologically indistinguishable species. Analyses based on partial mitochondrial *mtCOI* sequences determined at least 42 putative species (32–36) that include several genetic groups, here referred to as mitochondrial groups. All B. tabaci species complex members harbor a P-symbiont, the gamma-proteobacterium “*Candidatus* Portiera aleyrodidarum”(37). “*Ca.* Portiera” has a highly reduced genome (357 kbp) compared to the ones of evolutionarily-related free-living bacteria and cannot fully satisfy the metabolic need of its host since some of the essential amino acid biosynthetic pathways are incomplete. Moreover, the supply of vitamins and cofactors by “*Ca.* Portiera” seems to be restricted to carotenoids (38).

In addition to “*Ca.* Portiera,” seven S-symbionts have been identified in B. tabaci (genera *Arsenophonus*, *Cardinium*, *Fritschea*, *Hamiltonella*, *Hemipteriphilus*, *Rickettsia*, and *Wolbachia*), with up to four present in the same insect body (39). These S-symbionts colocalize with “*Ca.* Portiera” within bacteriocytes, and most of them can infect other tissues (6). They also have variable prevalence in B. tabaci populations. On the contrary, *Arsenophonus* and *Hamiltonella* are confined in the bacteriocytes and are almost fixed, but mutually exclusive, in the genetic groups in which they are found (39, 40).

Previous studies reported correlations between the S-symbiont composition and B. tabaci mitochondrial groups, both across (39) and within (40, 41) species. Moreover, different B. tabaci genetic groups have been found associated with particular biological and ecological features, such as geographic distribution (39) or host plant range (42, 43). These observations raise the possibility that S-symbionts may condition B. tabaci adaptation to its environment and its diversification, even though specific S-symbiont-host plant associations have never been documented to date in this species complex (39).

Analyses of B. tabaci S-symbiont genomes suggest that some of them could play a nutritional role in collaboration with the P-symbiont. For instance, the *Hamiltonella* genome encodes genes (*dapB*, *dapF*, *lysA*) involved in lysine biosynthesis that are lost or nonfunctional in “*Ca.* Portiera” (44). These genes are also present in the genome of B. tabaci, acquired from bacteria through ancient gene transfer events (45). These data suggest that the lysine biosynthesis could be achieved by the complementary interaction between either “*Ca.* Portiera” and *Hamiltonella*, “*Ca.* Portiera” and the insect host, or by a collaboration of the three of them. *In silico* genomic studies (44, 46) and experimental demonstrations (47, 48) also indicate that *Hamiltonella* in B. tabaci and *Arsenophonus* in Trialeurodes vaporariorum, a related whitefly species, can provide their hosts with B vitamins that “*Ca.* Portiera” can no longer provide. Conversely, other S-symbionts than *Hamiltonella* rely on the insect host or P- or other S-symbionts for the provision of nutrients (e.g., nonessential amino acids, nucleotides, and nucleosides) (49, 50). Therefore, S-symbionts are expected to impact their hosts’ dietary requirements, acting either as sources or sinks of essential metabolites. As the phloem sap composition varies between different plant species (51–54), S-symbionts could thereby positively or negatively influence the ability of insect hosts to exploit plants and then contribute to broaden or narrow their range of suitable host plants.

The aims of the present study were (i) to investigate the phenotypic and metabolic responses of B. tabaci and its symbiotic community to different host plants, and (ii) to decipher whether and how S-symbionts influence insect capacity to feed and produce offspring on those plants. Our results indicate that less suitable plants may constitute selective environments that particular cytotypes (designating insect line cytoplasmic features, including the mitochondrial genome and the intracellular symbiotic bacterial community) may help to exploit.

## RESULTS

### Plant amino acid content.

**FIG 1:**
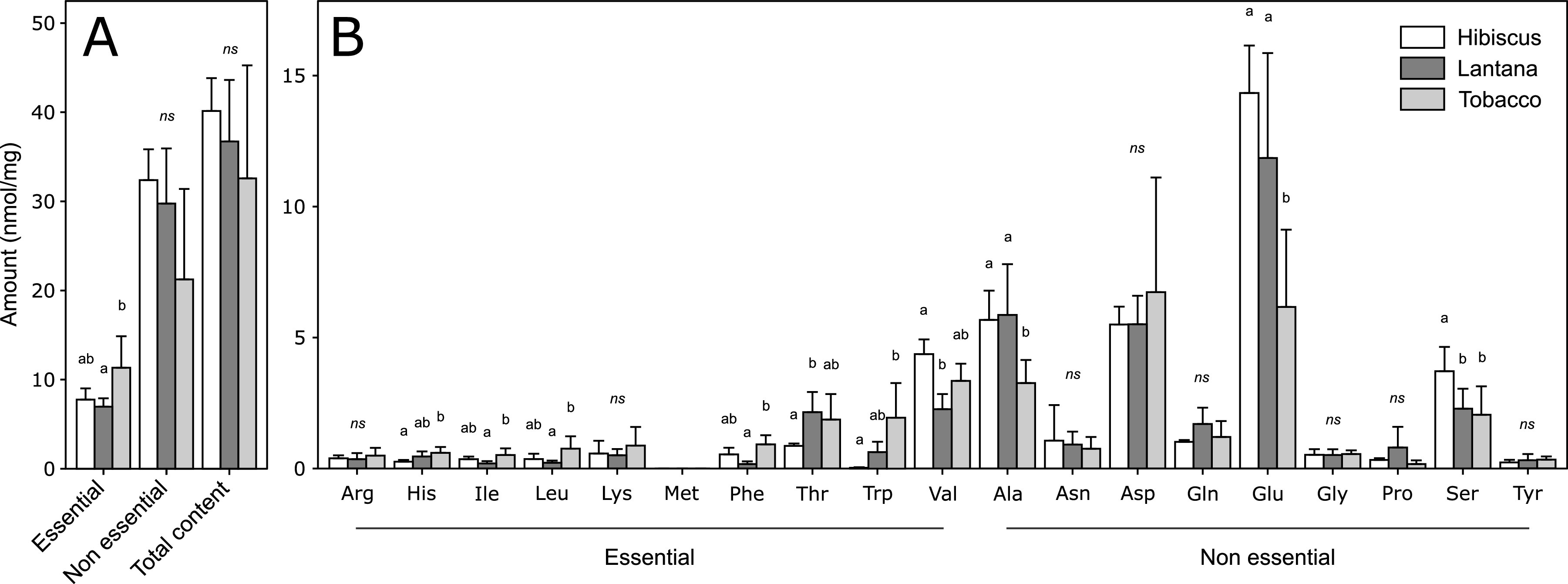
Foliar free amino acid content of three host plants of B. tabaci determined through HPLC analysis. (A) Essential, nonessential, and total amino acid contents; (B) individual amino acid content. The amino acid amount is expressed in nmol·mg^−1^ of dried tissue ± standard deviation (SD) (*n* = 5 replicates per plant). For each amino acid, comparisons between plants were performed by one-way ANOVA. Letters indicate significant differences between plants for a given amino acid (*P* < 0.05) determined by Tukey’s multiple comparisons analysis. ns, nonsignificant statistical differences. Ala, alanine; Arg, arginine; Asn, asparagine; Asp, aspartate; Gln, glutamine; Glu, glutamate; Gly, glycine; His, histidine; Ile, isoleucine; Leu, leucine; Lys, lysine; Met, methionine; Phe, phenylalanine; Pro, proline; Ser, serine; Thr, threonine; Trp, tryptophan; Tyr, tyrosine; Val, valine.

The plants used here, hibiscus (Hibiscus moscheutos), lantana (Lantana camara), and tobacco (Nicotiana tabacum), have been chosen because they are natural hosts for B. tabaci Mediterranean (MED) species. Their foliar free amino acid contents were measured through high-pressure liquid chromatography (HPLC) to test whether these plants have different nutritional properties. Amino acids were classified into two groups, amino acids considered essential (EAAs) (Arg, arginine; His, histidine; Ile, isoleucine; Leu, leucine; Lys, lysine; Met, methionine; Phe, phenylalanine; Thr, threonine; Trp, tryptophan; Val, valine) and nonessential (NEAAs) (Ala, alanine; Asn, asparagine; Asp, aspartate; Gln, glutamine; Glu, glutamate; Gly, glycine; Pro, proline; Ser, serine; Tyr, tyrosine) for B. tabaci (55). These three plants differed in their overall amount of EAAs (value of the *F* statistic [*F*_2,13_] = 5.25, *P = *0.023), which were greater in tobacco but similar in hibiscus and lantana. The opposite trend occurred for the total NEAA content (*F*_2,13_ = 3.34, *P = *0.070) (Fig. 1A). Looking at individual amino acids, the highest variation has been seen for 7 EAAs (His, Ile, Leu, Phe, Thr, Trp, and Val) and 3 NEAAs (Ala, Glu, and Ser) (Fig. 1B). Free amino acid amounts were analyzed using linear model (LM) (see details in “Statistical analyses”).

### Fecundity and hatching rate of B. tabaci lines on different plants.

**FIG 2:**
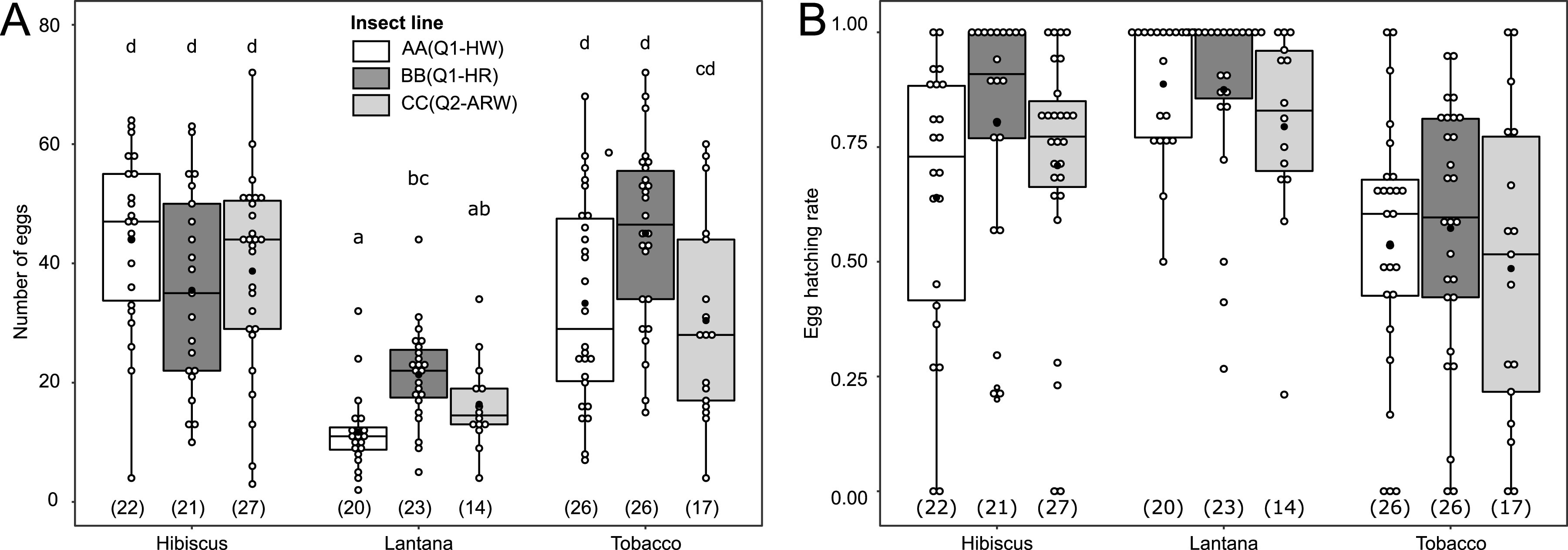
Fecundity and egg hatching rate of B. tabaci females belonging to the three lines used here and reared on hibiscus, lantana, or tobacco. (A) Number of eggs laid per female over 7 days. (B) Egg hatching rate after 10 days. Each dot represents one measure for one female, box length represents the interquartile range, and whiskers indicate the lowest and largest data points, excluding outliers. Black horizontal bars correspond to the median, and black dots correspond to the mean. “Insect line” represents nuclear genotype (cytotype); cytotype indicates mitochondrial group plus S-symbionts. S-symbionts include A, *Arsenophonus*; H, *Hamiltonella*; R, *Rickettsia*; and W, *Wolbachia*. Brackets beneath boxes indicate the number of replicates. To decipher the line-plant interaction in panel A, letters above the boxes indicate statistical groups determined by Tukey’s multiple-comparison test (*P* < 0.05).

Experiments were performed with three laboratory lines [namely, AA(Q1-HW), BB(Q1-HR), and CC(Q2-ARW)] from the B. tabaci MED species that belong to either the mitochondrial groups Q1 or Q2 and that are associated with different S-symbionts (Table 1). Whiteflies regularly reared on hibiscus in our laboratory conditions were transferred onto lantana and tobacco or were maintained on hibiscus for one generation. Female fecundity (number of oviposited eggs) and hatching rate on the same plant they developed on were used as performance indicators (Fig. 2; experimental design in Fig. S1A in the supplemental material). There was a significant effect of the interaction between the host plant species and the insect line on fecundity [χ^2^(4) = 18.05, *P = *0.001] (Fig. 2A). On hibiscus and tobacco, fecundity was similar and homogeneous between lines. However, fecundity was significantly lower on lantana, with differences between lines (*P < *0.05). Specifically, BB(Q1-HR) females laid 1.82 times more eggs (mean, 21.35; standard error [SE], 1.50) than AA(Q1-HW) females (mean, 11.7; SE, 1.50) (*P = *0.003), while line CC(Q2-ARW) had an intermediate fecundity (mean, 16.36; SE, 1.99). Regarding egg hatching rate, we found an additive effect of the plant species [χ^2^(2) = 53.42, *P < *0.001] and of the insect line [χ^2^(2) = 6.92, *P = *0.031], without interaction between these two factors [χ^2^(4) = 5.15, *P = *0.27] (Fig. 2B). The hatching rate differed between each plant species (*P < *0.05): the lowest was observed on tobacco, while it was higher on hibiscus and lantana. In general, the mean hatching rate was significantly higher in line BB(Q1-HR) than in CC(Q2-ARW) (*P = *0.019). AA(Q1-HW) had an intermediate hatching rate. Fecundity and hatching rates were analyzed with a mixed generalized linear model (GLM) with a negative binomial and a binomial error structure, respectively.

**TABLE 1:** Bemisia tabaci lines used in this study

Bemisia tabaci line	Nuclear genotype	Cytotype		Collection information		
		Mitochondrial group	Secondary endosymbionts^*a*^	Yr	Location	Plant
AA(Q1-HW)	AA	Q1	HW	2012	Tympaki, Greece	Eggplant (*Solanum* sp.)
BB(Q1-HR)	BB	Q1	HR	2012	Les Ponts-de-Cé, France	Mandevilla (*Mandevilla* sp.)
CC(Q2-ARW)	CC	Q2	ARW	2018	Lyon, France	Lantana (Lantana camara)

a HW, *Hamiltonella*, *Wolbachia*; HR, *Hamiltonella*, *Rickettsia*; ARW, *Arsenophonus*, *Rickettsia*, *Wolbachia*.

10.1128/mBio.00730-21.1 FIG S1 Experimental design used for phenotypic and physiological measures on hibiscus, lantana, and tobacco in “Collection of whiteflies for phenotypic and physiological measures on different plants” experiment (A) and “Collection of F_1_ hybrids or phenotypic and physiological measures on lantana” experiment (B). Individual fecundity measures, free amino acid analyses, and symbiont quantification were performed on F_1_ virgin females. The hatching rate was calculated as the ratio of the number of hatched eggs over the total number of eggs laid per F_1_ female. Download FIG S1, EPS file, 0.5 MB. Copyright © 2021 Benhamou et al. 2021 Benhamou et al. https://creativecommons.org/licenses/by/4.0/ This content is distributed under the terms of the Creative Commons Attribution 4.0 International license.

In order to determine whether the deleterious effect of lantana on female fecundity was due to the fact that females had spent their entire larval development on this plant, we also measured the fecundity of females developed on one plant (the donor) and subsequently transferred on the same plant or one of the two others (recipient plants). We tested all the possible combinations (experimental design in Fig. S2A) (Fig. S2B). Results indicated that, whatever the donor plant or the insect line used, there were significant differences in B. tabaci fecundity between the different recipient plants [χ^2^(2) = 142.92, *P < *0.001]: fecundity was lower on lantana than on hibiscus or tobacco (*P < *0.05) (Fig. S2C). There was also a significant interaction between the donor plant and the insect line [χ^2^(4) = 33.12, *P < *0.001]. Indeed, when larval development occurred on hibiscus or tobacco, fecundity on one of the three recipient plants was similar and homogeneous between lines. However, for lines AA(Q1-HW) and CC(Q2-ARW), fecundity was significantly lower when lantana was the donor plant, while it did not impact the line BB(Q1-HW) (Fig. S2D). Therefore, we demonstrated that both larval development, with differences between lines, and/or adult feeding on lantana negatively impact B. tabaci fecundity. Fecundity was analyzed with a GLM with a negative binomial error structure.

10.1128/mBio.00730-21.2 FIG S2 Effect of the plant where females developed or fed on (donor or recipient hostplant, respectively) on fecundity of B. tabaci females belonging to the three lines used here. (A) Schematic diagram of the plant switch experiment performed in this study. (B) Number of eggs laid per female over 7 days. Each dot represents one measure for a given female, box length represents the interquartile range, and whiskers indicate the lowest and largest data points, excluding outliers. Black horizontal bars correspond to the median, and black dots correspond to the mean. (C) Effect of the recipient plant on whiteflies mean fecundity (±95% confidence interval [CI]). (D) Effect of the interaction between the donor plant and insect line on whiteflies mean fecundity (±95% CI). Insect line represents nuclear genotype (cytotype); cytotype indicates mitochondrial group plus S-symbionts. S-symbionts include A, *Arsenophonus*; H, *Hamiltonella*; R, *Rickettsia*; and W, *Wolbachia*. Data were analyzed with a generalized linear model (GLM) (with a negative binomial error structure). In panels B and C, lowercase letters indicate statistical differences among recipient plants (B) or donor plants and insect lines combinations (C) (Tukey’s test, *P* < 0.05). Download FIG S2, EPS file, 0.6 MB. Copyright © 2021 Benhamou et al. 2021 Benhamou et al. https://creativecommons.org/licenses/by/4.0/ This content is distributed under the terms of the Creative Commons Attribution 4.0 International license.

### Amino acid content of B. tabaci lines on different plants.

**FIG 3:**
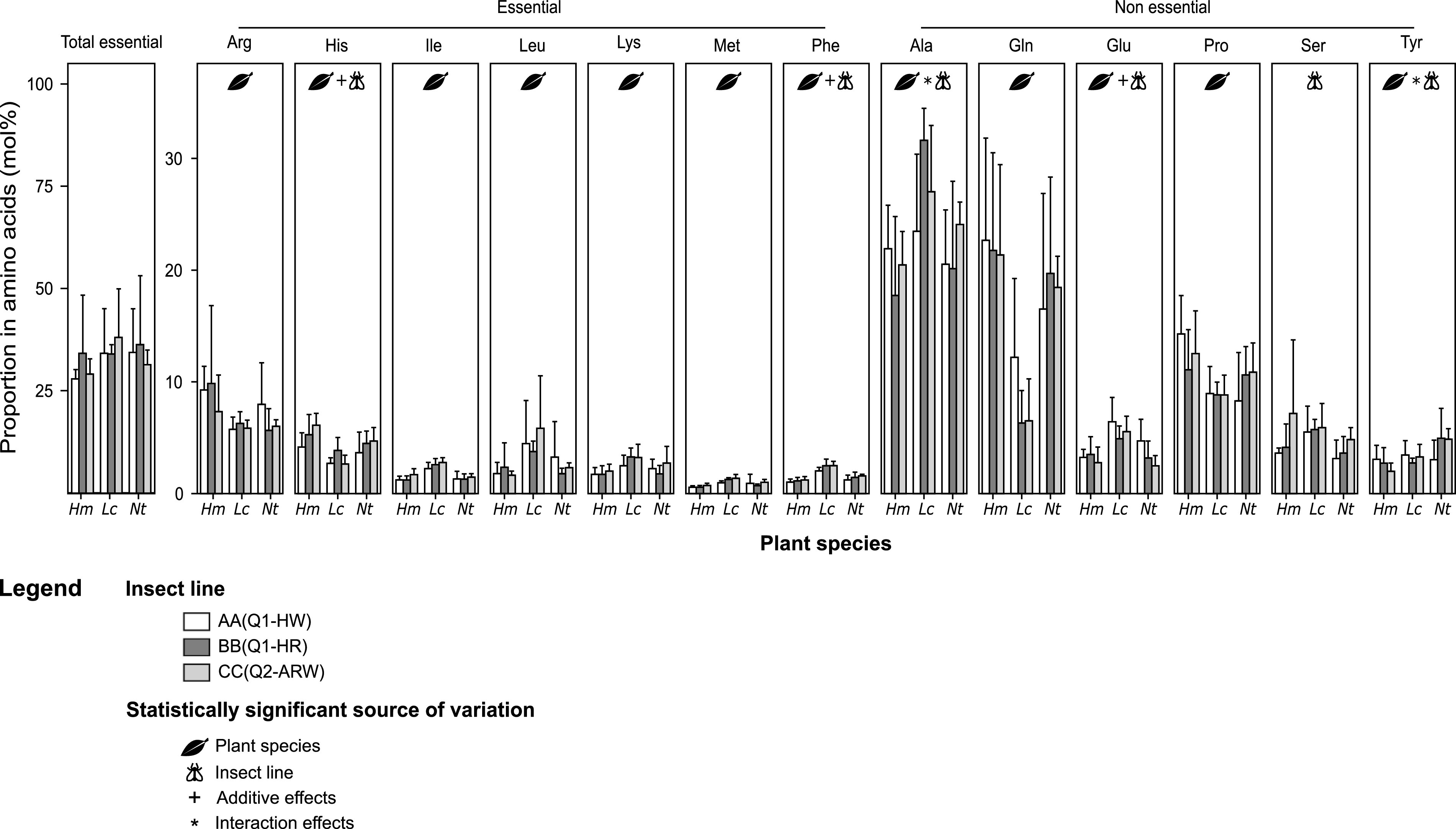
Total essential amino acid content (versus total nonessential amino acids) and individual free amino acid profiles of B. tabaci females belonging to the three lines used here and reared on hibiscus, lantana, or tobacco, as determined through HPLC analysis (*n* = 8) (mol%, mean ± SD). For the total essential amino acids and each individual amino acid, comparisons between plant species and insect lines were performed by two-way ANOVA. There was no difference between host plant or insect lines in the total essential amino acid proportion. Among the 19 amino acids analyzed, only those varying (*P* < 0.05; Table S2 in the supplemental material) are represented (see Table S1 for complete amino acid profile). Ala, alanine; Arg, arginine; Gln, glutamine; Glu, glutamate; His, histidine; Ile, isoleucine; Leu, leucine; Lys, lysine; Met, methionine; Phe, phenylalanine; Pro, proline; Ser, serine; Tyr, tyrosine. Hm, Hibiscus moscheutos (hibiscus); Lc, Lantana camara (lantana); Nt, Nicotiana tabacum (tobacco). “Insect line” represents nuclear genotype (cytotype); cytotype indicates mitochondrial group plus S-symbionts. S-symbionts include A, *Arsenophonus*; H, *Hamiltonella*; R, *Rickettsia*; and W, *Wolbachia*.

The free amino acid profile of B. tabaci lines was used as a proxy of their physiological state. HPLC analyses were performed on young females belonging to the three B. tabaci lines tested here and reared on hibiscus, lantana, or tobacco (Fig. 3; Tables S1 and S2). There was no statistical difference in the proportion of total EAAs (*versus* NEAAs) between plants or between insect lines (*F*_8,63_ = 0.86, *P = *0.56). Nevertheless, the proportions of Arg, Glu, Leu, Lys, Met, Gln, Pro, His, Ile, and Phe differed among whiteflies from the different plant species (value of the *F* statistic [*F*_2,63_] varied from 5.81 to 67.00 depending on the amino acid, *P < *0.05). Most of the differences occurred between whiteflies reared on lantana and the ones reared on hibiscus or tobacco, which have similar amino acid profiles (*P < *0.05) (Table S3). Specifically, Ile, Leu, Lys, Met, Phe, and Glu were more represented in insects reared on lantana (lantana/hibiscus mean fold change, Ile, 1.84; Leu, 2.43; Lys, 1.65; Met, 1.94; Phe, 2.08; Glu, 1.78). The opposite was found for glutamine (lantana/hibiscus mean fold change, 0.38). The amount of Glu, His, Ile, Ser, and Phe differed among insect lines (*F*_2,63_ varied from 3.26 to 5.24 depending on the amino acid, *P < *0.05). For Ala and Tyr, the effect of the host plant species differed between lines, as there was a significant interaction of the two factors (*F*_4,63_ varied from 3.05 to 3.29 depending on the amino acid, *P < *0.05). The amount of Ala was homogeneous between insect lines on hibiscus and tobacco, but was moderately higher in CC(Q2-ARW) and significantly higher in BB(Q1-HR) on lantana. The smallest amount of Tyr was found in CC(Q2-ARW) females reared on hibiscus, while the highest levels were found in BB(Q1-HR) and CC(Q2-ARW) females on tobacco. It had an intermediate level in every other line and plant combinations (Fig. 3). Free amino acid proportions in insects were analyzed using LM.

10.1128/mBio.00730-21.3 TABLE S1 Free amino acid profiles of B. tabaci females belonging to the three lines used here and reared on hibiscus, lantana, or tobacco, as determined through HPLC analysis (*n* = 8) (mean mol%, mean ± SE). Download Table S1, DOCX file, 0.02 MB. Copyright © 2021 Benhamou et al. 2021 Benhamou et al. https://creativecommons.org/licenses/by/4.0/ This content is distributed under the terms of the Creative Commons Attribution 4.0 International license.

10.1128/mBio.00730-21.4 TABLE S2 Two-way ANOVA analysis of the free amino acid profile of B. tabaci females belonging to the three lines used here and reared on hibiscus, lantana, or tobacco, as determined through HPLC analysis (summarized in Fig. 3). Download Table S2, DOCX file, 0.02 MB. Copyright © 2021 Benhamou et al. 2021 Benhamou et al. https://creativecommons.org/licenses/by/4.0/ This content is distributed under the terms of the Creative Commons Attribution 4.0 International license.

10.1128/mBio.00730-21.5 TABLE S3 *P* values from multiple comparisons (Tukey’s pairwise comparison) of the free amino acid content in B. tabaci females between plant species. Data from each insect line were compared jointly. For each amino acid, analyses were carried out only when the plant factor had a statistically significant effect on the amino acid content in two-way ANOVA (Table S2; Fig. 3). Download Table S3, DOCX file, 0.01 MB. Copyright © 2021 Benhamou et al. 2021 Benhamou et al. https://creativecommons.org/licenses/by/4.0/ This content is distributed under the terms of the Creative Commons Attribution 4.0 International license.

### Symbiont density.

**FIG 4:**
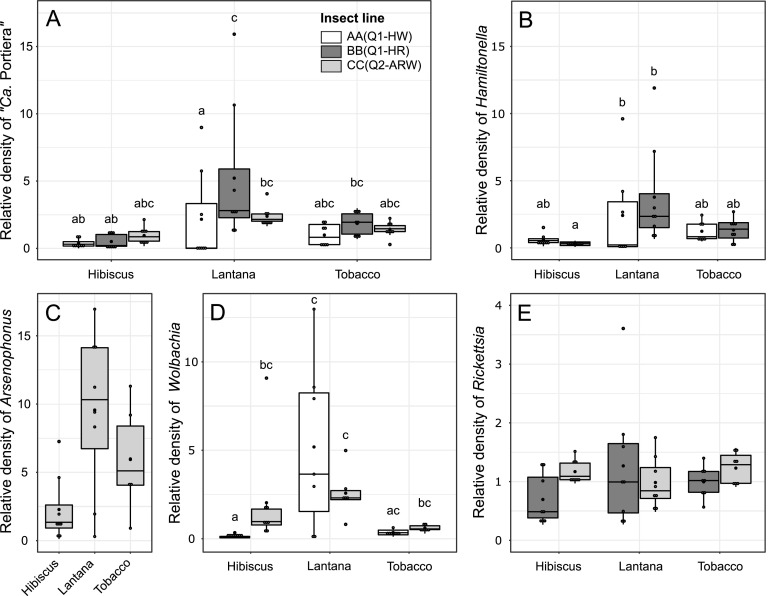
Relative symbiotic density in B. tabaci females belonging to the three lines used here and reared on hibiscus, lantana, or tobacco (number of bacterial cells per host cell, *n* = 8). (A) “*Ca.* Portiera”; (B) *Hamiltonella*; (C) *Arsenophonus*; (D) *Wolbachia*; (E) *Rickettsia*. Each dot represents one measure for one female, box length represents the interquartile range, and whiskers indicate the lowest and largest data points, excluding outliers. Black horizontal bars (within the box) correspond to the median, and black dots correspond to the mean. “Insect line” represents nuclear genotype (cytotype); cytotype indicates mitochondrial group plus S-symbionts. S-symbionts include A, *Arsenophonus*; H, *Hamiltonella*; R, *Rickettsia*; and W, *Wolbachia*. Letters above the boxes indicate statistical groups among line and host plant combinations (Tukey’s test, *P* < 0.05).

We found a significant effect of the interaction between the host plant and the insect line on the relative amount (number of bacterial cells per host cell) of “*Ca.* Portiera” (*F*_2,62_ = 3.25, *P = *0.018), *Hamiltonella* (*F*_2,40_ = 4.26, *P = *0.021), and *Wolbachia* (*F*_2,38_ = 6.37, *P = *0.0041) (Fig. 4A, B, and D). In BB(Q1-HR) and CC(Q2-ARW) lines, “*Ca.* Portiera” density tended to be higher on lantana than on hibiscus and tobacco. Alternatively, the line AA(Q1-HW) exhibited the same modest “*Ca.* Portiera” density on the three plants (Fig. 4A). The same pattern was observed for *Hamiltonella* and *Wolbachia* (Fig. 4B and D), as the densities of these two S-symbionts were correlated with the density of “*Ca.* Portiera” (Spearman rho rank test, *Hamiltonella*, ρ = 0.84, *P < *0.001; *Wolbachia*, ρ = 0.52, *P < *0.001). The same trend was observed for the relative amount of *Arsenophonus* in line CC(Q2-ARW) (*F*_2,19_ = 3.48, *P = *0.052) (Fig. 4C). The relative amount of *Rickettsia* in insects remained at the same level regardless of the host plant they developed on (*F*_2,41_ = 1.04, *P = *0.36) or the insect line (*F*_1,41_ = 4.03, *P = *0.051), with no interaction between the two factors (*F*_2,41_ = 1.97, *P = *0.15) (Fig. 4E). Globally, for each insect line, all symbionts (except *Rickettsia*) had the same densities on hibiscus and tobacco but tended to increase on lantana. Symbiont density was analyzed with LM.

### Fecundity and hatching rate of hybrids on lantana according to the cytotype.

**FIG 5:**
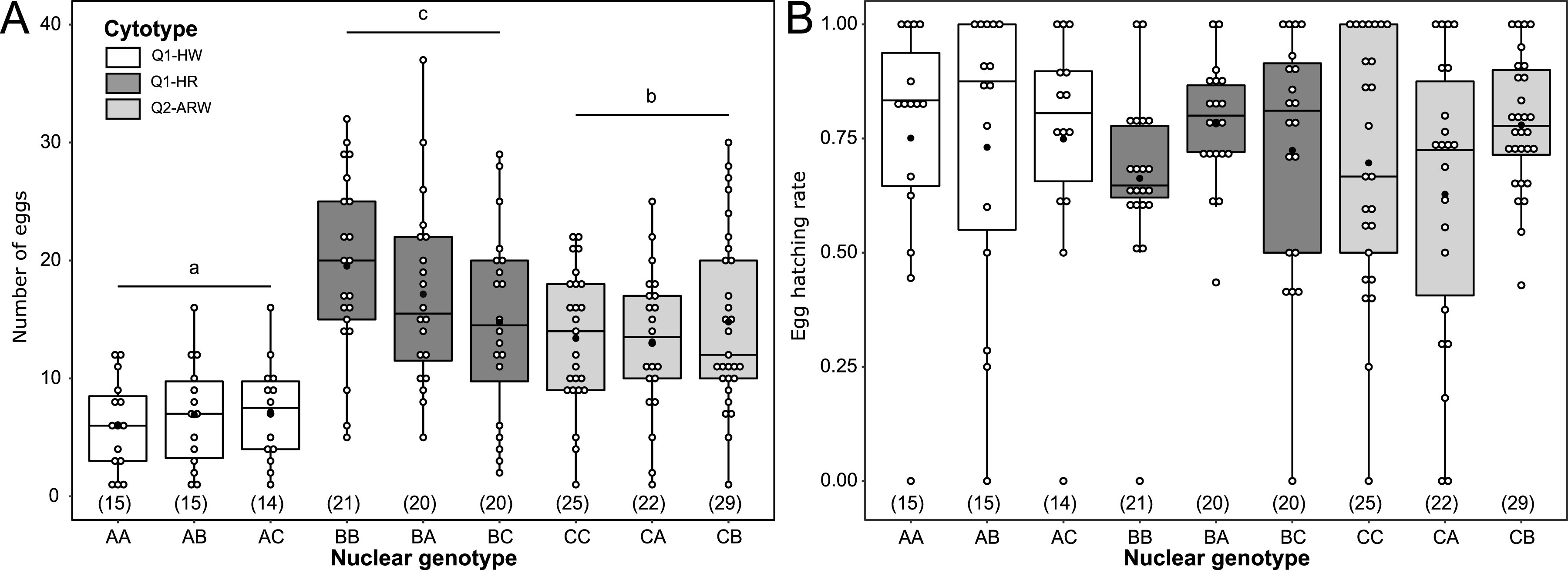
Effect of the insects’ genotype and cytotype on B. tabaci females’ performance on lantana. (A) Number of eggs laid per female over 7 days; (B) egg hatching rate after 10 days. Each dot represents one measure for one female, box length represents the interquartile range, and whiskers indicate the lowest and largest data points, excluding outliers. Black horizontal bars correspond to the median, and black dots correspond to the mean. Brackets beneath boxes indicate the number of replicates. Letters above the boxes indicate statistical groups among nuclear genotypes and cytotypes combinations (Tukey’s test, *P* < 0.05). All individuals are F_1_ females obtained from crosses of the laboratory lines AA(Q1-HW), BB(Q1-HR), or CC(Q2-ARW). “F_1_ females” represents the nuclear genotype (cytotype). For nuclear genotype, letters represent the maternally (first) and the paternally (second) inherited nuclear genotype. Cytotype indicates mitochondrial group plus S-symbionts. S-symbionts include A, *Arsenophonus*; H, *Hamiltonella*; R, *Rickettsia*; and W, *Wolbachia*.

In order to disentangle whether differences in insects’ performance on lantana were due to females’ genotype or cytotype, we performed crosses between parental lines to obtain F_1_ hybrids (Table 2, experimental design in Fig. S1B). Crosses produced females bearing the same nuclear genotype but differing in their cytotypes [e.g., AB(Q1-HW) and BA(Q1-HR)], or the opposite, i.e., females harboring the same cytotype but differing in their genotype [e.g., AA(Q1-HW), AB(Q1-HW), and AC(Q1-HW)]. There was an overall effect of insect cytotype on fecundity [χ^2^(2) = 76.22, *P < *0.001], but, for each cytotype, there was no difference between genotypes [χ^2^(6) = 4.66, *P = *0.59]. Females with cytotype Q1-HW laid the lowest number of eggs, whatever the nuclear genotype (Fig. 5A). We detected no influence of the insect cytotype [χ^2^(2) = 0.034, *P = *0.76] or nuclear genotype [χ^2^(6) = 0.44, *P = *0.30] on the egg hatching rate (Fig. 5B). Fecundity and hatching rates were analyzed with a mixed GLM with a negative binomial and a binomial error structure, respectively. These results show an influence of the cytotype and a limited effect of the genotype on female fecundity on lantana.

**TABLE 2:** Nuclear genotype and cytotype of B. tabaci F_1_ females obtained from crosses of F_0_ individuals from three parental lines^*a*^

Father (F_0_) line	Mother (F_0_) line		
	AA(Q1-HW)	BB(Q1-HR)	CC(Q2-ARW)
A(Q1-HW)	AA(Q1-HW)	BA(Q1-HR)	CA(Q2-ARW)
B(Q1-HR)	AB(Q1-HW)	BB(Q1-HR)	CB(Q2-ARW)
C(Q2-ARW)	AC(Q1-HW)	BC(Q1-HR)	CC(Q2-ARW)

a “F_1_ females” represents the nuclear genotype (cytotype). For nuclear genotype, letters represent the maternally (first) and the paternally (second) inherited nuclear genotype. Cytotype indicates mitochondrial group plus S-symbionts. S-symbionts include A, *Arsenophonus*; H, *Hamiltonella*; R, *Rickettsia*; and W, *Wolbachia*. F_0_ male genotypes are indicated by only one letter, as they are haploid.

### Free amino acid content in hybrids on lantana.

**FIG 6:**
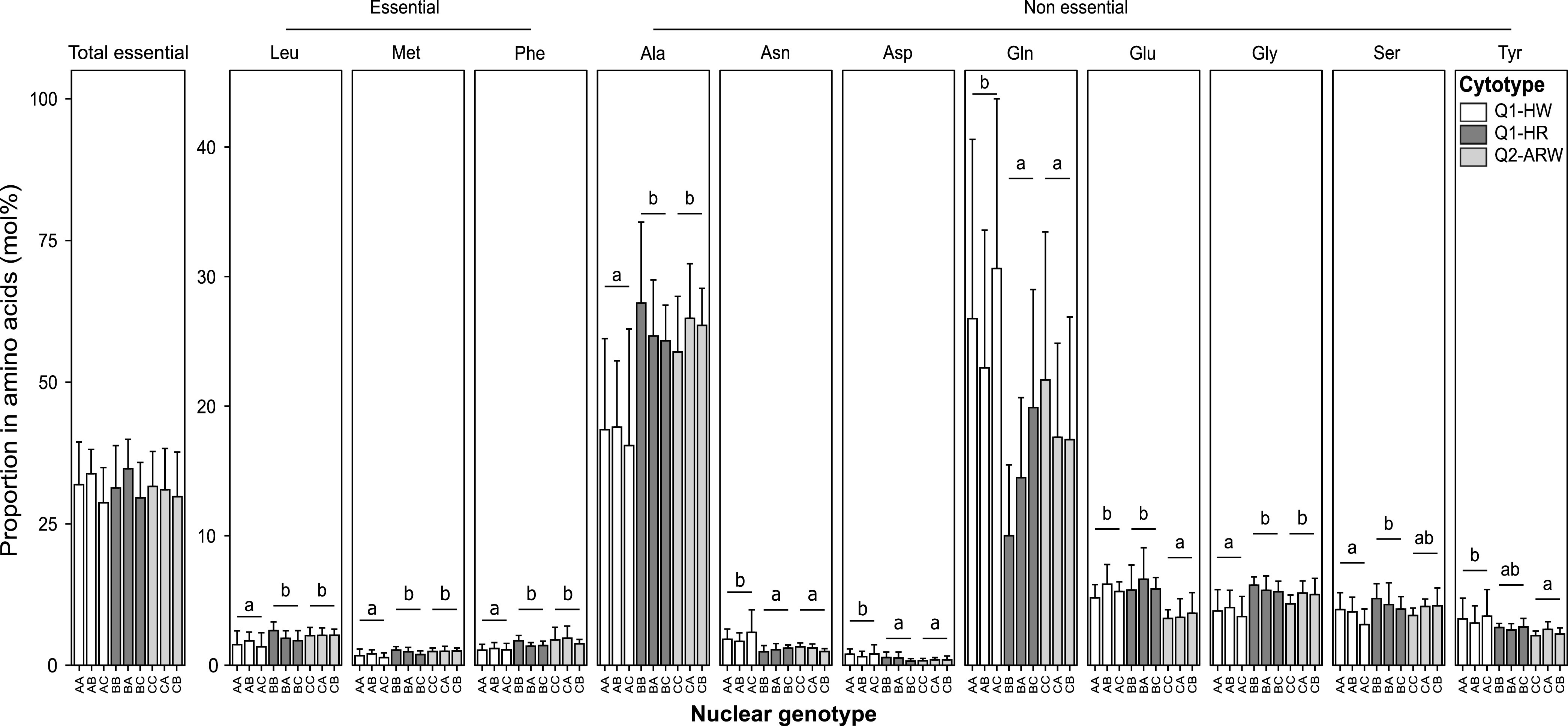
Total essential amino acid proportion (versus total nonessential amino acids) and individual free amino acid profile in B. tabaci females on lantana, determined through HPLC analysis on individual females (*n* = 8) (mol%, mean ± SD). For the total essential amino acids and each amino acid, comparisons between groups were performed by two-way ANOVA. Letters indicate statistical differences between cytotypes (*P* < 0.05) determined by Tukey's multiple comparisons analysis (see Table S6 in the supplemental material for *P* values). Among the 19 amino acids analyzed, only those varying (*P* < 0.05) are represented (see Table S4 for complete amino acid profile). Ala, alanine; Asn, asparagine; Asp, aspartate; Gln, glutamine; Glu, glutamate; Gly, glycine; Leu, leucine; Met, methionine; Phe, phenylalanine; Ser, serine; Tyr, tyrosine. All individuals are F_1_ hybrid females obtained from the crosses of laboratory lineages AA(Q1-HW), BB(Q1-HR), or CC(Q2-ARW). “F_1_ females” represents the nuclear genotype (cytotype). For nuclear genotype, letters represent the maternally (first) and the paternally (second) inherited nuclear genotype. Cytotype indicates mitochondrial group plus S-symbionts. S-symbionts include A, *Arsenophonus*; H, *Hamiltonella*; R, *Rickettsia*; and W, *Wolbachia*.

To determine whether the cytotype had an impact on the insect amino acid metabolism, we performed HPLC analyses on F_1_ females (Fig. 6; Tables S4 and S5). There was no statistical difference in the proportion of total EAAs (versus total NEAAs) either between insect cytotypes or genotypes (*F*_8,63_ = 0.70, *P = *0.69). However, 3 out of the 10 EAAs (Leu, Met, and Phe) and 8 out of the 9 NEAAs (Ala, Asn, Asp, Gln, Glu, Gly, Ser, and Tyr) significantly differed between cytotypes (value of the F statistic [*F*_2,63_] varied from 3.45 to 17.77 depending on the amino acid, *P < *0.05). The insect’s genotype had no significant effect on the percentage of each individual amino acid (value of the F statistic [*F*_6,63_] varied from 0.21 to 1.71 depending on the amino acid, *P > *0.05). Most differences occurred between the Q1-HW cytotype and the two other cytotypes (*P < *0.05; Table S6). Specifically, Leu, Met, Phe, Ala, and Gly were less abundant, and Asn and Gln were more abundant in Q1-HW females. Free amino acid proportions in insects were analyzed using LM. These results indicate that whiteflies’ amino acid profile mainly depends on the insect cytotype.

10.1128/mBio.00730-21.6 TABLE S4 Free amino acid profile in B. tabaci females on lantana, determined through HPLC analysis on individual females (*n* = 8) (mean mol% ± SE). Download Table S4, DOCX file, 0.02 MB. Copyright © 2021 Benhamou et al. 2021 Benhamou et al. https://creativecommons.org/licenses/by/4.0/ This content is distributed under the terms of the Creative Commons Attribution 4.0 International license.

10.1128/mBio.00730-21.7 TABLE S5 Two-way ANOVA table investigating the effect of insects’ cytotype and genotype on the free amino acid content in B. tabaci females on lantana. Download Table S5, DOCX file, 0.02 MB. Copyright © 2021 Benhamou et al. 2021 Benhamou et al. https://creativecommons.org/licenses/by/4.0/ This content is distributed under the terms of the Creative Commons Attribution 4.0 International license.

10.1128/mBio.00730-21.8 TABLE S6 *P* values from multiple comparisons (Tukey’s pairwise comparison) of the free amino acid content in B. tabaci females on lantana between cytotypes. For each amino acid, analyses were carried out only when the cytotype factor had a significant effect on the amino acid content in two-way ANOVA (Fig. 6; Table S5). Download Table S6, DOCX file, 0.01 MB. Copyright © 2021 Benhamou et al. 2021 Benhamou et al. https://creativecommons.org/licenses/by/4.0/ This content is distributed under the terms of the Creative Commons Attribution 4.0 International license.

10.1128/mBio.00730-21.9 TABLE S7 Plants parts used for the free amino acid analysis of plants leaves. Download Table S7, DOCX file, 0.01 MB. Copyright © 2021 Benhamou et al. 2021 Benhamou et al. https://creativecommons.org/licenses/by/4.0/ This content is distributed under the terms of the Creative Commons Attribution 4.0 International license.

### P- and S-symbiont densities in hybrids.

**FIG 7:**
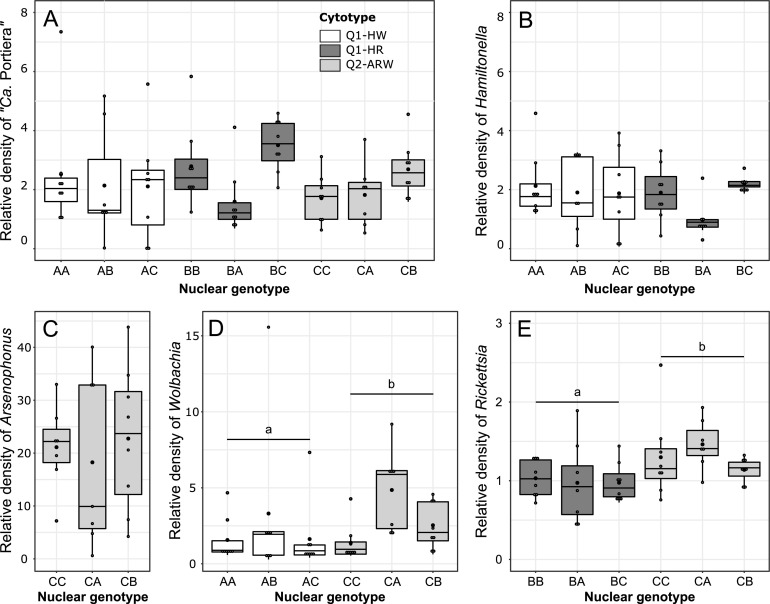
Relative symbiotic densities in B. tabaci females on lantana (number of bacterial cells per host cell, *n* = 8). (A) “*Ca.* Portiera”; (B) *Hamiltonella*; (C) *Arsenophonus*; (D) *Wolbachia*; (E) *Rickettsia*. Each dot represents one measure for one female, box length represents the interquartile range, and whiskers indicate the lowest and largest data points, excluding outliers. Black horizontal bars (within boxes) correspond to the median, and black dots correspond to the mean. All individuals are F_1_ females obtained from crosses of the laboratory lines AA(Q1-HW), BB(Q1-HR), or CC(Q2-ARW). “F_1_ females” represents the nuclear genotype (cytotype). For nuclear genotype, letters represent the maternally (first) and the paternally (second) inherited nuclear genotype. Cytotype indicates mitochondrial group plus S-symbionts. S-symbionts include A, *Arsenophonus*; H, *Hamiltonella*; R, *Rickettsia*; and W, *Wolbachia*. Letters above the boxes indicate statistical groups among nuclear genotype and cytotype combinations (Tukey’s test, *P* < 0.05).

To determine whether, apart from differences in S-symbiont infection status, the phenotypic and physiological variations observed between cytotypes were correlated with different symbiont densities, we analyzed symbiotic density in F_1_ females on lantana (Fig. 7). There was neither a significant difference between cytotypes in the relative amount of “*Ca.* Portiera” (*F*_2,61_ = 2.34, *P = *0.11) and *Hamiltonella* (*F*_1,41_ = 3.25, *P = *0.079), nor between genotypes within each cytotype for “*Ca.* Portiera” (*F*_6,61_ = 1.36, *P = *0.25), *Hamiltonella* (*F*_4,41_ = 1.28, *P = *0.29), and *Arsenophonus* (*F*_2,19_ = 1.00, *P = *0.39) (Fig. 7A to C). Alternatively, there were significant differences between cytotypes in the relative amount of *Wolbachia* (*F*_1,40_ = 4.80, *P = *0.034; 1.74 times more abundant in Q2-ARW than in Q1-HW) and *Rickettsia* (*F*_1,41_ = 0.95, *P = *0.0037; 1.35 times more abundant in Q2-ARW than in Q1-HR), but not between genotypes within each cytotype for *Wolbachia* (*F*_4,40_ = 2.416, *P = *0.065) and *Rickettsia* (*F*_4,41_ = 4.12, *P = *0.55) (Fig. 7D and E). Symbiont density was analyzed using LM.

## DISCUSSION

In the present study, we showed that the foliar free amino acid content of three natural host plants for B. tabaci MED species, hibiscus, lantana, and tobacco, is nutritionally unbalanced, dominated by nonessential amino acids (NEAAs) (e.g., Glu, Asp, and Ala) with a limited amount of essential amino acids (EAAs). Our data corroborate previous reports for tobacco (56), but data we obtained for hibiscus and lantana are new. They are in agreement with data available for the phloem sap of a large spectrum of plants (51, 52, 54). Interestingly, these three plant species differ in their free amino acid composition: they thus constitute a nutrient-contrasted environment that may lead to different selective pressures for whiteflies and their symbionts.

Plant species influenced all the traits of B. tabaci we measured. Importantly, whitefly oviposition was significantly reduced on lantana, indicating that this plant is relatively less favorable for MED Q1 and Q2 than hibiscus or tobacco. This result confirms data previously reported in western Africa, for which oviposition on lantana of MED Q1 individuals was reduced compared to cotton (57). In this study, we also demonstrated that, whatever the plant females were transferred on for oviposition, their fecundity was reduced if they had developed on lantana. Taken together, our results indicate that lantana is a particularly challenging host for B. tabaci complex members. This could explain why, in the field, populations reported on this plant are restricted to MED (39, 41, 58), including in areas where other B. tabaci species co-occur, such as the close relative polyphagous MEAM1 (Middle East Asia minor 1) species (59). It is tempting to associate this impairment with the lowest nutritional quality of lantana, which contains the smallest amount of the four EAAs, Ile, Leu, Phe, and Val, among the three plants considered in our study. Besides the fact that these amino acids are considered being essential for the development of all metazoans, studies specifically focusing on whiteflies have shown that small amounts of Ile in artificial diets were correlated with a reduction of their oviposition ability (60). Moreover, experiments conducted on aphids have suggested that plant amino acid content could influence their reproduction (61).

Differences in free amino acid profiles of B. tabaci were observed between individuals reared on lantana and the ones that developed on hibiscus and tobacco, indicating that the insect physiological state varies between an unfavorable and a favorable plant. In whiteflies reared on hibiscus and tobacco, the most represented amino acids were Gln, Ala, and Pro (NEAAs) and Arg (EAA). Similar observations have been reported in MED individuals reared on tomato and poinsettia (62, 63) and in the close relative species, MEAM1, on cotton, cucumber, eggplant, and tomato (63–65), which are also considered favorable hosts for MED and MEAM1 species (43, 66–68). In whiteflies reared on lantana, five EAAs (Ile, Leu, Lys, Met, and Phe) and one NEAA (Glu) were more represented than in whiteflies reared on the two other plants. Others, such as glutamine, were less represented. Glutamine plays a central role in amino acid metabolism, as it is the main precursor of the EAAs synthesized by the nutritional symbionts (55, 69). Therefore, the specific amino acid profile found on lantana may indicate an increased production of EAAs, supported by the consumption of the glutamine pool, possibly compensating for specific nutrient deficiencies in the plant. Indeed, some of the most represented EAAs in insects were also less abundant in lantana than in the other two plants. Similarly, MEAM1 individuals reared on low-amino-acid-content plants show a large reduction in glutamine pool regarding other amino acids (64). Alternatively, an increased EAA production in insects may result from larger amounts of EAA precursors in the host plant (i.e., NEAAs). However, there seems to be no correlation between the amount of specific NEAAs in hibiscus, lantana, and tobacco and the relative proportion of specific EAAs in whiteflies reared on those plants. For instance, neither the total NEAAs nor each individual NEAA content was specifically higher in lantana, while insects reared on lantana showed higher proportions of Ile, Leu, Lys, Met, and Phe than other plants. Thus, the increased EAAs production in insects reared on lantana is more likely the result of the insect’s metabolic demand, rather than the plant’s NEAA content.

Previous studies suggest that plant amino acid content influences symbiont density in B. tabaci. A decrease of EAAs abundance in plants has been associated with an increase of “*Ca.* Portiera” relative amount (65), possibly to meet the host metabolic demand. In our study, both P- and S-symbionts densities were higher on lantana. The observed increase in “*Ca.* Portiera” density in whiteflies reared on lantana may compensate for specific EAA deficiencies of this plant. This hypothesis is supported by the observation, reported above, that high “*Ca.* Portiera” relative amounts also correlate with an increase of the EAA content in whiteflies reared on lantana. Overall, these findings are consistent with data available for the aphid Acyrthosiphon pisum, where an antibiotic treatment targeting the P-symbiont B. aphidicola has resulted in a decrease of EAAs and an accumulation of EAA precursors in the insect body (70). Similar to the increase of “*Ca.* Portiera” density, higher S-symbiont densities in whiteflies reared on lantana could also be important to meet the insect metabolic need on an unfavorable plant, as some of those S-symbionts retained nutritional functions (44–46, 71). However, whiteflies have poor fecundity when reared on lantana. One possible explanation is that high symbiont relative amounts, consistent with an increase of the EAA content in insect body, may not fully satisfy the insect metabolic needs. Moreover, other factors than nutrition, such as mechanical defenses and defensive compounds produced by the plant and not known yet, may also affect whitefly performance.

In addition to the differences in insect fecundity, hatching rate, free amino acid profile, and symbiont densities between plant species, differences had also been observed between whitefly lines on lantana, which was not the case for the two other plants. Our analysis of hybrid females (that share the same nuclear background but different cytotypes) showed that they present different fecundities and free amino acid profiles on lantana. On the contrary, hybrid females harboring the same cytotype but different nuclear genotypes did not show such differences. These data indicate that the cytotype determines both insects’ fecundity and free amino acid profile. The whiteflies’ cytotype comprises different genetic entities, the host mitochondrial genome and the symbionts. A substantial contribution of the mitochondrial genome to plant resources utilization appears unlikely. Indeed, previous analyses showed that mitochondrial DNA (mtDNA) divergence between MED Q1 and Q2 populations is low, ranging from 0.15 to 1.09% (72). Additionally, Q1-HW and Q1-HR, supposedly the closest cytotypes regarding their mtDNA, are also the most different phenotypically in their responses to plants. Differences related to genetic and/or gene expression variability of the P-symbiont among cytotypes also seems unlikely, given the low genetic variability and the constant metabolic capabilities of “*Ca.* Portiera” within the B. tabaci species complex (38, 44, 73–75) and the almost complete loss in the “*Ca.* Portiera” genome of elements for gene expression regulation (that seems to be ensured by the whitefly host) (73). Conversely, considering their genomic capabilities and based on the results presented above, S-symbionts are promising candidates to explain the differences observed between the three B. tabaci lines considered here. We also do not exclude the possibility that these observations result from nongenetic maternal effects (76).

Fecundity was higher in Q1-HR and Q2-ARW than in Q1-HW whiteflies. These two cytotypes also showed higher proportions of the EAAs Leu, Met, and Phe, while the opposite was observed for the NEAA Gln. As discussed above, this amino acid signature suggests higher EAA production in Q1-HR and Q2-ARW cytotypes. Therefore, insect fecundity could be mediated by a potential direct or indirect impact of S-symbionts on the amino acid metabolism of their hosts. In B. tabaci, S-symbionts could complement the P-symbiont and directly contribute to EAAs biosynthesis, as some of them (e.g., *Hamiltonella* and *Rickettsia*) retained biosynthesis genes lost in “*Ca.* Portiera” (44, 71). Metabolic complementation between P- and S-symbionts may be facilitated in this insect model by their colocalization in the same bacteriocytes (6).

The overall effect of the cytotype on whiteflies may depend on the infection by a single S-symbiont species or result as the net effect of the entire symbiotic community. For example, *Rickettsia* is associated with both cytotypes Q1-HR and Q2-ARW with similar fecundity and free amino acid profile. Interestingly, *Rickettsia* from MEAM1 conserved genes involved in the biosynthesis pathways of EAAs Phe (*tyrB*), Val, Leu, and Ile (*ilvE*) (71). Therefore, *Rickettsia* could benefit its host by contributing to the production of these EAAs. Interestingly, infection by *Rickettsia* has been associated with fitness benefits in MEAM1 natural populations from the United States (77). However, it was not the case in populations from Israel, suggesting geographic differences in the interaction of this symbiont and its host (78). Additionally, fitness benefits associated with the infection by *Rickettsia* populations from the United States were no longer observed years later (79), possibly due to the interaction of the symbiont with the host’s nuclear genetic background (80). Under the hypothesis that *Rickettsia* provides fitness benefits to its insect host on lantana, this S-symbiont is expected to be found in higher prevalence in B. tabaci natural populations colonizing lantana than other plants. However, in a survey done in Burkina Faso (West Africa), a lower prevalence of *Rickettsia* was reported in MED Q3 populations colonizing lantana (26.6%) than tobacco (90%) (41), but additional studies are needed to tackle this question.

In conclusion, this work shows that host plant utilization by B. tabaci is determined by the insect cytotype, most likely by the S-symbiont composition. These results raise two immediate questions. The first addresses the individual roles of S-symbionts. As discussed above, the effect of the cytotype may result from the infection by one or multiple S-symbiont species, and future research should investigate the contribution of each single S-symbiont to the host phenotype. In this perspective, recent studies have made significant advances in manipulating B. tabaci symbiotic community, using antibiotic and thermal treatments (47, 48) that could be actually used to eliminate specific S-symbionts and determine the effect of this treatment on phenotypic and physiological parameters of the insect host. The second question relates to the underlying mechanisms by which S-symbionts contribute to their host phenotype. We propose that they may contribute to broadening the range of their suitable host plants through their metabolic contribution. Future investigations should consider the use of artificial diets of controlled amino acid composition (60, 70) to accurately determine the influence of S-symbionts on their host’s amino acid dietary requirements.

## MATERIALS AND METHODS

### Insects.

Three B. tabaci lines were used in this study, namely, AA(Q1-HW), BB(Q1-HR), and CC(Q2-ARW) (Table 1). The first two letters (AA, BB, and CC) arbitrarily designate female diploid nuclear genotype. Characters within brackets represent the insect cytotype [e.g., (Q1-HW)], composed of the mitochondrial group (Q1 or Q2) and its associated symbiotic community (A, *Arsenophonus*; H, *Hamiltonella*; R, *Rickettsia*; W, *Wolbachia*). These lines were reared in stock cages on hibiscus plants in climate-controlled rooms at 26 ± 1°C and 60% relative humidity with a 14-h light/10-h dark photoperiod. Whitefly mitochondrial group and symbiont infection status were checked on eight individuals per line before and after the experiments, which ensured that the expected cytotype was correct. The mitochondrial group was identified by PCR-restriction fragment length polymorphism (RFLP), based on the use of the mitochondrial cytochrome oxidase 1 gene sequence (*mtCO1*) as described in Henri et al. (81). Whitefly symbiont infection status was determined using the quantitative PCR (qPCR) protocol described below.

### Plants.

Plants were grown in insect-free climate-controlled rooms at 26 ± 1°C and 70% relative humidity with a 14-h light/10-h dark photoperiod. Hibiscus (Hibiscus moscheutos, Malvaceae) and tobacco (Nicotiana tabacum, Solanaceae) were grown from seeds. Lantana (Lantana camara, Verbenaceae) was propagated from plant cuttings in pots using 75:25 (vol/vol) mix of potting soil and vermiculite. Plants were watered twice a week. Fully developed leaves (younger than 2-month-old plants) were used to perform phenotypic and physiological measures on whiteflies.

### Collection of whiteflies for phenotypic and physiological measures on different plants.

We explored the phenotypic and metabolic responses of B. tabaci and its symbionts to different host plants by performing transfers from the rearing plant hibiscus to lantana, tobacco, or hibiscus itself (experimental design shown in Fig. S1A in the supplemental material). Synchronized whitefly females were obtained by allowing adults from the hibiscus stock cages (F_0_ generation) to mate and oviposit for 7 days on either hibiscus, lantana, or tobacco leaf disks fixed on their adaxial face on a 1-cm layer of 2% agar (Sigma-Aldrich) in petri dishes (90 mm) stored under the same conditions as the stock cages. This procedure was repeated three times per plant and per whitefly line, accounted as “batches” in the following statistical analyses. Petri dishes were monitored every day: after the first emergence, all adults were removed, and 5 h later, newly emerged adults were collected (F_1_ generation). The sex of the emerged individuals was determined based on their genitalia by observation under a stereomicroscope (Leica EZ4; Leica Microsystems GmbH, Wetzlar, Germany). As newly emerged adults do not immediately reproduce, this procedure ensured female virginity, which was confirmed by the absence of females in the offspring (B. tabaci is a haplodiploid species; only fertilized eggs engender females). Newly emerged females were isolated on new leaf disks of the same plant species they developed on. Young females (0 to 7 days) were collected and stored at −80°C for subsequent HPLC analyses. Others were left to oviposit for 7 days. After 7 days, the number of oviposited eggs of F_1_ females was recorded under a stereomicroscope, and individuals were stored at −80°C for the determination of symbiont density. Ten days after the female removal, the number of larvae was counted to estimate the hatching rate (number of larvae/number of oviposited eggs). Females that had not laid eggs were discarded, and 14 to 27 valid measures of fecundity and hatching rate per condition were obtained.

We also tested whether the insect development on one of the three plants tested here had an impact on female fecundity. Briefly, F_0_ adults regularly reared on hibiscus were allowed to lay eggs on hibiscus, lantana, or tobacco (donor host plant). Upon emergence, F_1_ virgin females were transferred on the same plant they develop on or on one of the two others (recipient plants) All donor and recipient host plant combinations were tested (experimental design shown in Fig. S2A).

### Collection of F_1_ hybrids for phenotypic and physiological measures on lantana.

We performed all possible crosses between the three lines, AA(Q1-HW), BB(Q1-HR), and CC(Q2-ARW) (Table 2). Virgin females and males from the parental lines reared on hibiscus were collected from leaf disks. Crosses were done on lantana (90-mm petri dish) by bringing together groups of 15 females and 15 males, and mated females were left for 1 week on the same lantana leaf disk to oviposit. Each cross was performed in three replicates, accounted as batches in the following statistical analyses. Upon emergence, F_1_ virgin females were isolated on fresh lantana leaf disks (55-mm petri dish) for measures of fecundity, hatching rate, symbiont density, and free amino acid content by following the same protocol as described above (“Collection of whiteflies for phenotypic and physiological measures on different plants”) (experimental design shown in Fig. S1B). For each cross, 8 F_1_ females were screened for all S-symbionts present in F_0_ parents, which ensured that the F_1_ females' infection status was the same as their mother.

### Free amino acid extraction from plant leaves.

Free amino acid contents in hibiscus, lantana, and tobacco were assessed from leaves of controlled age (Table S7). Fresh leaves were cut into pieces, lyophilized, and mechanically crushed (TissueLyser; Qiagen, Hilden, Germany) using stainless steel balls (1.4 mm) for 1 min at 20 Hz. Then, 4 mg of tissues were suspended in 20 μl of ultrapure water with a known amount of norvaline (10 nmol) used as an internal standard. Free amino acids were extracted from 200 μl of this crude homogenate with trichloroacetic acid (TCA; 5% [wt/vol] final concentration), maintained at room temperature for 2 h, vortexed every 30 min, and then centrifuged (10,000 × *g* for 10 min at 4°C). TCA was eliminated from the supernatant by chloroform/water partition (three successive extractions with 400 μl of chloroform), and the final aqueous supernatant was dried under vacuum. Samples were stored at −20°C and then mixed with 100 μl of ultrapure water for amino acid analysis. Five biological replicates, consisting of leaves from different plants, were performed for each host plant species.

### Whitefly free amino acid extraction.

Free amino acids in whiteflies were extracted from single individual insects adapting a protocol initially developed for detection in aphid embryos (82). An absolute analysis of amino acid content, as the one obtained from plant leaves, was impossible, as we were not able to weigh living individuals, even with a precision balance, because of their very low weight (around 30 μg) and their constant movements. Samples were mechanically crushed (TissueLyser; Qiagen) with stainless steel balls (1.4 mm) for 1 min at 20 Hz in 100 μl of ultrapure water with a known quantity of norvaline (1 nmol) used as the internal standard. Free amino acids were extracted from 75 μl of this crude homogenate with 300 μl of ethanol (EtOH; 80% [wt/vol] final concentration), maintained at room temperature for 2 h, vortexed every 30 min, and then centrifuged (10,000 × *g* for 10 min at 4°C), and the supernatant was dried under vacuum. Samples were stored at −20°C and then mixed with 5 μl of ultrapure water for amino acid analysis. Four biological replicates were performed for each condition.

### Amino acid HPLC analysis.

Amino acid quantification was performed using HPLC (Agilent 1100; Agilent Technologies) with a guard cartridge and a reverse-phase C_18_ column (Zorbax Eclipse AAA; 3.5 μm, 150 by 4.6 mm; Agilent Technologies), according to the procedure specifically developed for this system (83) and subsequently adapted to aphid tissues (82). The derivatization process, at room temperature, was automated using the Agilent 1313A autosampler. Detection was performed by a fluorescence detector set at 340 and 450 nm of excitation and emission wavelengths, respectively (266/305 nm for proline). Under these conditions, oxidations can lead to several cysteine forms, which does not allow its accurate detection and quantification, so only 19 amino acids were quantified. For this quantification, norvaline was used as the internal standard, and the response factor of each amino acid was determined using a 250-μM standard mix of amino acids. The software used for the analysis was ChemStation for LC three-dimensional systems.

### Symbiont quantification.

Symbiont density was measured on single insects using qPCR. Briefly, DNA was extracted from each individual using the NucleoSpin 96 tissue kit (Macherey-Nagel GmbH, Düren, Germany). Samples were crushed with sterile stainless steel balls (1.4 mm) in 60 μl of lysis buffer added with 8 μl of proteinase K (22 mg/ml) by a TissueLyser (Qiagen). DNA was extracted following the instructions from the manufacturer, eluted in 100 μl of buffer, and stored at −20°C until use. Each qPCR consisted of 5 μl SYBR Green 2× mastermix (Bio-Rad, Hercules, USA), 2 μl nuclease-free water, 2 μl DNA sample, and 0.5 μl of forward and reverse primer (500 nM final concentration). The following genes, *16S rRNA* for *Arsenophonus*, *dnaK* for *Hamiltonella*, *gltA* for *Rickettsia*, *ftsZ* for *Wolbachia*, and *β-actin* for B. tabaci, were used for data normalization (Table 3). Quantifications included eight biological replicates with two technical replicates for each sample. Samples were run on a CFX-96 real-time PCR machine and analyses were done using the CFX Manager software v3.1 (Bio-Rad, Hercules, USA). The relative amount of each symbiont was normalized using *β-actin* (normalized relative quantity [NRQ]). For each gene, two samples were used as calibrators to check for interplate variability. Interplate calibration was performed when interplate variability between calibrators was higher than 0.5 quantification cycle (*C_q_*), which was the case for *Arsenophonus 16S rRNA*.

**TABLE 3:** qPCR primers used in this study

Organism	Gene	Primer	Primer sequence	Fragment size (bp)	Hybridization temp (°C)	Reference
Bemisia tabaci	β-actin	wf-Bactin-F	5′-TCTTCCAGCCATCCTTCTTG-3′	130	63	88
		wf-Bactin-R	5′-CGGTGATTTCCTTCTGCATT-3′	130	63	88
“*Ca.* Portiera”	16S	Port73-F	5′-GTGGGGAATAACGTACGG-3′	193	60	89
		Port266-R	5′-CTCAGTCCCAGTGTGGCTG-3′	193	60	89
*Rickettsia*	GltA	glt375-F	5′-TGGTATTGCATCGCTTTGGG-3′	199	60	89
		glt574-R	5′-TTTCTTTAAGCACTGCAGCACG-3′	199	60	89
*Wolbachia*	FtsZ	F2	5′-TTGCAGAGCTTGGACTTGAA-3′	400	55	90
		R2	5′-CATATCTCCGCCACCAGTAA-3′	400	55	90
*Hamiltonella*	dnaK	dnaK-F	5′-GGTTCAGAAAAAAGTGGCAG-3′	155	60	91
		dnaK-R	5′-CGAGCGAAAGAGGAGTGAC-3′	155	60	91
*Arsenophonus*	16S	ArsF3	5′-GTCGTGAGGAARGTGTTARGGTT-3′	765	63	92
		ArsR3	5′-CCTYTATCTCTAAAGGMTTCGCTGGATG-3′	765	63	92

### Statistical analyses.

Data analysis was performed using R software (R Development Core Team; http://www.R-project.org). When necessary, the normality of the residual error and the homogeneity of the variance were checked to ensure that the modeling approach was appropriate. HPLC data (free amino acid amount in nmol·mg^−1^ for the plant leaves and free amino acid proportions (mol%) in insects) were analyzed using analysis of variance (ANOVA) for each amino acid. Amino acid proportions in insects were analyzed using proportions as continuous variables without transformation. Phenotypic measures (fecundity and hatching rate) were analyzed by a mixed generalized linear model (GLMM) (with a log link and a negative binomial or a binomial error structure, respectively). For the phenotypic measures on the three host plants, the plant species and the insect line were set as fixed effects. For the phenotypic measures of F_1_ hybrids on lantana, the fixed effects included the insect cytotype and its genotype; the latter was integrated into the model as a fixed nested effect within the cytotype. For both experiments, experimental batches were set as a random effect. Symbiont NRQs were calculated using the EasyqpcR package (84) based on the method proposed by Hellemans and collaborators (85) and analyzed by a linear model (LM) applied to log-transformed data. Multiple comparison analyses were performed by Tukey’s test (86) to investigate differences between groups using the emmeans function of the emmeans package (87).

### Data availability.

All data sets generated and analyzed in the present study are available in Zenodo at https://zenodo.org/record/5520874#.YWsOJhrMJPY.
