# Supplementary material for: Cytotype Affects the Capability of the Whitefly Bemisia tabaci MED Species To Feed and Oviposit on an Unfavorable Host Plant
Source: mBio. 2021 Nov 16;12(6):e00730-21. doi: 10.1128/mBio.00730-21 (PMC8593682; doi:10.1128/mBio.00730-21)
Supplement: TABLE S1 [file mbio.00730-21-st001.docx]

**Table S1**. Free amino acid profiles of *B. tabaci* females from three lines on hibiscus, lantana and tobacco, determined through HPLC analysis (n=8) (mean mol%, mean ± SE).

| **Plant** | **Hibiscus** | | | | | | | | |  | **Lantana** | | | | | | | | |  | **Tobacco** | | | | | | | | |
| --- | --- | --- | --- | --- | --- | --- | --- | --- | --- | --- | --- | --- | --- | --- | --- | --- | --- | --- | --- | --- | --- | --- | --- | --- | --- | --- | --- | --- | --- |
| **Line** | **AA(Q1-HW)** | | | **BB(Q1-HR)** | | | **CC(Q2-ARW)** | | |  | **AA(Q1-HW)** | | | **BB(Q1-HR)** | | | **CC(Q2-ARW)** | | |  | **AA(Q1-HW)** | | | **BB(Q1-HR)** | | | **CC(Q2-ARW)** | | |
| **Ala** | 21.92 | ± | 1.38 | 17.73 | ± | 2.50 | 20.47 | ± | 1.06 |  | 23.48 | ± | 2.44 | 31.62 | ± | 1.01 | 27.02 | ± | 2.10 |  | 20.53 | ± | 1.72 | 20.14 | ± | 2.76 | 24.09 | ± | 0.71 |
| **Arg** | 9.27 | ± | 0.75 | 9.85 | ± | 2.47 | 7.34 | ± | 1.16 |  | 5.75 | ± | 0.39 | 6.28 | ± | 0.37 | 5.85 | ± | 0.25 |  | 7.98 | ± | 1.32 | 5.65 | ± | 0.69 | 6.02 | ± | 0.21 |
| **Asn** | 0.57 | ± | 0.11 | 0.91 | ± | 0.30 | 1.30 | ± | 0.39 |  | 1.45 | ± | 0.35 | 1.20 | ± | 0.20 | 1.18 | ± | 0.24 |  | 0.71 | ± | 0.22 | 0.71 | ± | 0.19 | 0.93 | ± | 0.15 |
| **Asp** | 0.79 | ± | 0.13 | 1.16 | ± | 0.24 | 0.97 | ± | 0.19 |  | 1.76 | ± | 0.37 | 1.19 | ± | 0.22 | 1.45 | ± | 0.36 |  | 1.02 | ± | 0.18 | 1.26 | ± | 0.38 | 0.83 | ± | 0.18 |
| **Gln** | 22.67 | ± | 3.23 | 21.76 | ± | 3.09 | 21.36 | ± | 2.86 |  | 12.20 | ± | 2.49 | 6.31 | ± | 1.03 | 6.51 | ± | 1.33 |  | 16.52 | ± | 3.66 | 19.71 | ± | 3.05 | 18.46 | ± | 0.98 |
| **Glu** | 3.24 | ± | 0.25 | 3.51 | ± | 0.56 | 2.76 | ± | 0.49 |  | 6.43 | ± | 0.77 | 4.91 | ± | 0.40 | 5.55 | ± | 0.49 |  | 4.71 | ± | 0.69 | 3.18 | ± | 0.54 | 2.48 | ± | 0.32 |
| **Gly** | 4.98 | ± | 0.52 | 5.54 | ± | 0.61 | 4.33 | ± | 0.46 |  | 6.05 | ± | 0.61 | 6.19 | ± | 0.51 | 5.52 | ± | 0.59 |  | 10.70 | ± | 3.91 | 4.46 | ± | 0.54 | 6.13 | ± | 0.46 |
| **His** | 4.16 | ± | 0.45 | 5.27 | ± | 0.64 | 6.12 | ± | 0.37 |  | 2.70 | ± | 0.18 | 3.86 | ± | 0.41 | 2.64 | ± | 0.28 |  | 3.66 | ± | 0.65 | 4.49 | ± | 0.39 | 4.70 | ± | 0.43 |
| **Ile** | 1.22 | ± | 0.12 | 1.22 | ± | 0.13 | 1.70 | ± | 0.19 |  | 2.23 | ± | 0.20 | 2.59 | ± | 0.19 | 2.78 | ± | 0.15 |  | 1.31 | ± | 0.24 | 1.29 | ± | 0.17 | 1.47 | ± | 0.11 |
| **Leu** | 1.79 | ± | 0.36 | 2.35 | ± | 0.77 | 1.64 | ± | 0.13 |  | 4.47 | ± | 1.36 | 3.76 | ± | 0.33 | 5.85 | ± | 1.68 |  | 3.26 | ± | 1.13 | 1.78 | ± | 0.17 | 2.32 | ± | 0.15 |
| **Lys** | 1.72 | ± | 0.22 | 1.72 | ± | 0.27 | 2.01 | ± | 0.22 |  | 2.49 | ± | 0.34 | 3.28 | ± | 0.28 | 3.23 | ± | 0.42 |  | 2.23 | ± | 0.30 | 1.77 | ± | 0.26 | 2.73 | ± | 0.53 |
| **Met** | 0.56 | ± | 0.05 | 0.57 | ± | 0.05 | 0.73 | ± | 0.07 |  | 0.96 | ± | 0.06 | 1.27 | ± | 0.05 | 1.37 | ± | 0.12 |  | 0.91 | ± | 0.29 | 0.71 | ± | 0.05 | 1.01 | ± | 0.09 |
| **Phe** | 1.03 | ± | 0.10 | 1.13 | ± | 0.11 | 1.21 | ± | 0.11 |  | 2.03 | ± | 0.11 | 2.49 | ± | 0.21 | 2.50 | ± | 0.14 |  | 1.22 | ± | 0.15 | 1.45 | ± | 0.17 | 1.58 | ± | 0.05 |
| **Pro** | 14.29 | ± | 1.22 | 11.08 | ± | 1.27 | 12.53 | ± | 1.35 |  | 8.95 | ± | 0.86 | 8.84 | ± | 0.40 | 8.83 | ± | 0.63 |  | 8.29 | ± | 1.53 | 10.62 | ± | 0.90 | 10.87 | ± | 0.93 |
| **Ser** | 3.63 | ± | 0.16 | 4.14 | ± | 0.74 | 7.18 | ± | 2.33 |  | 5.51 | ± | 0.82 | 5.73 | ± | 0.32 | 5.90 | ± | 0.76 |  | 3.13 | ± | 0.59 | 3.62 | ± | 0.53 | 4.83 | ± | 0.38 |
| **Thr** | 1.41 | ± | 0.14 | 2.91 | ± | 1.28 | 1.99 | ± | 0.57 |  | 3.64 | ± | 1.30 | 2.29 | ± | 0.15 | 4.54 | ± | 2.08 |  | 3.61 | ± | 1.07 | 2.17 | ± | 0.67 | 2.37 | ± | 0.36 |
| **Trp** | 0.77 | ± | 0.09 | 0.55 | ± | 0.07 | 0.79 | ± | 0.05 |  | 0.83 | ± | 0.13 | 0.75 | ± | 0.06 | 0.68 | ± | 0.06 |  | 0.68 | ± | 0.10 | 0.68 | ± | 0.08 | 0.76 | ± | 0.05 |
| **Tyr** | 3.06 | ± | 0.44 | 2.72 | ± | 0.49 | 2.00 | ± | 0.26 |  | 3.45 | ± | 0.45 | 2.74 | ± | 0.15 | 3.28 | ± | 0.40 |  | 3.03 | ± | 0.62 | 4.95 | ± | 0.94 | 4.87 | ± | 0.33 |
| **Val** | 2.92 | ± | 0.32 | 5.89 | ± | 2.68 | 3.58 | ± | 0.17 |  | 5.61 | ± | 2.06 | 4.69 | ± | 0.23 | 5.34 | ± | 1.06 |  | 6.50 | ± | 2.46 | 11.35 | ± | 7.24 | 3.57 | ± | 0.21 |
| Ala: Alanine; Arg: Arginine; Asn: Asparagine; Asp: Aspartate; Gln: Glutamine; Glu: Glutamate; Gly: Glycine; His: Histidine; Ile: Isoleucine; Leu: Leucine; Lys: Lysine; Met: Methionine; Phe: Phenylalanine; Pro: Proline; Ser: Serine; Thr: Threonine; Trp: Tryptophan; Tyr: Tyrosine; Val: Valine. Insect line key: Nuclear genotype(Cytotype); Cytotype: Mitochondrial group+S-symbionts; S-symbionts: A: *Arsenophonus*, H: *Hamiltonella*, R: *Rickettsia*, W: *Wolbachia.* | | | | | | | | | | | | | | | | | | | | | | | | | | | | | |
