# Supplementary material for: Cytotype Affects the Capability of the Whitefly Bemisia tabaci MED Species To Feed and Oviposit on an Unfavorable Host Plant
Source: mBio. 2021 Nov 16;12(6):e00730-21. doi: 10.1128/mBio.00730-21 (PMC8593682; doi:10.1128/mBio.00730-21)
Supplement: TABLE S2 [file mbio.00730-21-st002.docx]

**Table S2.** Two-way ANOVA analysis of the free amino acid profile of *B. tabaci* females from three lines on hibiscus, lantana, and tobacco, determined through HPLC analysis (summarized on **Fig. 3**).

| **Amino_Acid** | **Parameter** | **Df** | **Sum Sq** | **Mean Sq** | **F value** | **Pr(>F)** |  |  |
| --- | --- | --- | --- | --- | --- | --- | --- | --- |
| Asp | Plant | 2 | 3.433 | 1.716 | 3.067 | 0.054 |  |  |
|  | Line | 2 | 0.221 | 0.111 | 0.198 | 0.821 |  |  |
|  | Plant:Line | 4 | 2.380 | 0.595 | 1.063 | 0.382 |  |  |
| Glu | Plant | 2 | 86.655 | 43.327 | 19.670 | <0.001 | *** |  |
|  | Line | 2 | 18.911 | 9.455 | 4.293 | 0.018 | ** |  |
|  | Plant:Line | 4 | 13.425 | 3.356 | 1.524 | 0.206 |  |  |
| Asn | Plant | 2 | 3.073 | 1.537 | 2.999 | 0.057 |  |  |
|  | Line | 2 | 0.733 | 0.367 | 0.715 | 0.493 |  |  |
|  | Plant:Line | 4 | 2.064 | 0.516 | 1.007 | 0.411 |  |  |
| Ser | Plant | 2 | 41.631 | 20.815 | 2.886 | 0.063 |  |  |
|  | Line | 2 | 46.957 | 23.478 | 3.255 | 0.045 | * |  |
|  | Plant:Line | 4 | 24.788 | 6.197 | 0.859 | 0.493 |  |  |
| Gln | Plant | 2 | 2370.048 | 1185.024 | 21.898 | <0.001 | *** |  |
|  | Line | 2 | 36.139 | 18.069 | 0.334 | 0.717 |  |  |
|  | Plant:Line | 4 | 191.258 | 47.814 | 0.884 | 0.479 |  |  |
| His | Plant | 2 | 54.093 | 27.047 | 17.012 | <0.001 | *** |  |
|  | Line | 2 | 16.233 | 8.116 | 5.105 | 0.009 | ** |  |
|  | Plant:Line | 4 | 11.599 | 2.900 | 1.824 | 0.135 |  |  |
| Gly | Plant | 2 | 55.266 | 27.633 | 1.762 | 0.180 |  |  |
|  | Line | 2 | 56.702 | 28.351 | 1.808 | 0.172 |  |  |
|  | Plant:Line | 4 | 117.979 | 29.495 | 1.881 | 0.125 |  |  |
| Thr | Plant | 2 | 23.198 | 11.599 | 1.338 | 0.270 |  |  |
|  | Line | 2 | 3.563 | 1.781 | 0.205 | 0.815 |  |  |
|  | Plant:Line | 4 | 35.698 | 8.924 | 1.029 | 0.399 |  |  |
| Arg | Plant | 2 | 109.305 | 54.652 | 5.808 | 0.005 | ** |  |
|  | Line | 2 | 20.023 | 10.012 | 1.064 | 0.351 |  |  |
|  | Plant:Line | 4 | 34.118 | 8.530 | 0.906 | 0.466 |  |  |
| Ala | Plant | 2 | 717.290 | 358.645 | 12.706 | <0.001 | *** |  |
|  | Line | 2 | 43.549 | 21.774 | 0.771 | 0.467 |  |  |
|  | Plant:Line | 4 | 371.351 | 92.838 | 3.289 | 0.016 | * |  |
| Tyr | Plant | 2 | 35.563 | 17.782 | 8.795 | <0.001 | *** |  |
|  | Line | 2 | 1.060 | 0.530 | 0.262 | 0.770 |  |  |
|  | Plant:Line | 4 | 24.690 | 6.173 | 3.053 | 0.023 | * |  |
| Val | Plant | 2 | 111.480 | 55.740 | 0.881 | 0.420 |  |  |
|  | Line | 2 | 127.388 | 63.694 | 1.006 | 0.371 |  |  |
|  | Plant:Line | 4 | 162.394 | 40.598 | 0.641 | 0.635 |  |  |
| Met | Plant | 2 | 4.079 | 2.040 | 18.477 | <0.001 | *** |  |
|  | Line | 2 | 0.694 | 0.347 | 3.142 | 0.050 |  |  |
|  | Plant:Line | 4 | 0.546 | 0.137 | 1.237 | 0.304 |  |  |
| Trp | Plant | 2 | 0.043 | 0.022 | 0.414 | 0.663 |  |  |
|  | Line | 2 | 0.132 | 0.066 | 1.255 | 0.292 |  |  |
|  | Plant:Line | 4 | 0.283 | 0.071 | 1.348 | 0.262 |  |  |
| Phe | Plant | 2 | 19.332 | 9.666 | 67.007 | <0.001 | *** |  |
|  | Line | 2 | 1.511 | 0.755 | 5.236 | 0.008 | ** |  |
|  | Plant:Line | 4 | 0.302 | 0.076 | 0.524 | 0.718 |  |  |
| Ile | Plant | 2 | 21.750 | 10.875 | 46.065 | <0.001 | *** |  |
|  | Line | 2 | 1.987 | 0.994 | 4.209 | 0.019 | * |  |
|  | Plant:Line | 4 | 0.621 | 0.155 | 0.657 | 0.624 |  |  |
| Leu | Plant | 2 | 103.516 | 51.758 | 8.503 | <0.001 | *** |  |
|  | Line | 2 | 5.727 | 2.863 | 0.470 | 0.627 |  |  |
|  | Plant:Line | 4 | 23.576 | 5.894 | 0.968 | 0.431 |  |  |
| Lys | Plant | 2 | 17.249 | 8.625 | 9.963 | <0.001 | *** |  |
|  | Line | 2 | 3.452 | 1.726 | 1.994 | 0.145 |  |  |
|  | Plant:Line | 4 | 3.834 | 0.959 | 1.107 | 0.361 |  |  |
| Pro | Plant | 2 | 180.523 | 90.261 | 9.941 | <0.001 | *** |  |
|  | Line | 2 | 3.881 | 1.941 | 0.214 | 0.808 |  |  |
|  | Plant:Line | 4 | 69.675 | 17.419 | 1.918 | 0.118 |  |  |
| Ala: Alanine; Arg: Arginine; Asn: Asparagine; Asp: Aspartate; Gln: Glutamine; Glu: Glutamate; Gly: Glycine; His: Histidine; Ile: Isoleucine; Leu: Leucine; Lys: Lysine; Met: Methionine; Phe: Phenylalanine; Pro: Proline; Ser: Serine; Thr: Threonine; Trp: Tryptophan; Tyr: Tyrosine; Val: Valine. Significance key: P<0.001 '***', P<0.01 '**', P<0.05 '*'. | | | | | | | | |
