# Supplementary material for: Cytotype Affects the Capability of the Whitefly Bemisia tabaci MED Species To Feed and Oviposit on an Unfavorable Host Plant
Source: mBio. 2021 Nov 16;12(6):e00730-21. doi: 10.1128/mBio.00730-21 (PMC8593682; doi:10.1128/mBio.00730-21)
Supplement: TABLE S3 [file mbio.00730-21-st003.docx]

| **Amino acid** | **Contrast** | **P-value** |  |  |
| --- | --- | --- | --- | --- |
| Glu | Hibiscus - Lantana | <0.001 | *** |  |
|  | Hibiscus - Tobacco | 0.775 |  |  |
|  | Lantana - Tobacco | <0.001 | *** |  |
| Gln | Hibiscus - Lantana | <0.001 | *** |  |
|  | Hibiscus - Tobacco | 0.197 |  |  |
|  | Lantana - Tobacco | <0.001 | *** |  |
| His | Hibiscus - Lantana | <0.001 | *** |  |
|  | Hibiscus - Tobacco | 0.042 | * |  |
|  | Lantana - Tobacco | 0.004 | ** |  |
| Arg | Hibiscus - Lantana | 0.006 | ** |  |
|  | Hibiscus - Tobacco | 0.034 | * |  |
|  | Lantana - Tobacco | 0.784 |  |  |
| Met | Hibiscus - Lantana | <0.001 | *** |  |
|  | Hibiscus - Tobacco | 0.024 | ** |  |
|  | Lantana - Tobacco | 0.004 | * |  |
| Phe | Hibiscus - Lantana | <0.001 | *** |  |
|  | Hibiscus - Tobacco | 0.026 | * |  |
|  | Lantana - Tobacco | <0.001 | *** |  |
| Ile | Hibiscus - Lantana | <0.001 | *** |  |
|  | Hibiscus - Tobacco | 0.987 |  |  |
|  | Lantana - Tobacco | <0.001 | *** |  |
| Leu | Hibiscus - Lantana | <0.001 | *** |  |
|  | Hibiscus - Tobacco | 0.742 |  |  |
|  | Lantana - Tobacco | 0.007 | * |  |
| Lys | Hibiscus - Lantana | <0.001 | *** |  |
|  | Hibiscus - Tobacco | 0.260 |  |  |
|  | Lantana - Tobacco | 0.017 | * |  |
| Pro | Hibiscus - Lantana | <0.001 | *** |  |
|  | Hibiscus - Tobacco | 0.008 | ** |  |
|  | Lantana - Tobacco | 0.452 |  |  |
| Significance key : P<0.001 '***', P<0.01 '**', P<0.05 '*' | | | | |

**Table S3**. P-values from multiple comparisons (Tukey’s pairwise comparison) of the free amino acid content in *B. tabaci* females between plant species. Data from each insect line were compared jointly. For each amino acid, analyses were carried out only when the plant factor had a statistically significant effect on the amino acid content in two-way ANOVA (**Table S2**, **Fig. 3)**
