# Supplementary material for: Cytotype Affects the Capability of the Whitefly Bemisia tabaci MED Species To Feed and Oviposit on an Unfavorable Host Plant
Source: mBio. 2021 Nov 16;12(6):e00730-21. doi: 10.1128/mBio.00730-21 (PMC8593682; doi:10.1128/mBio.00730-21)
Supplement: TABLE S4 [file mbio.00730-21-st004.docx]

|  | **AA(Q1-HW)** | | | **AB(Q1-HW)** | | | **AC(Q1-HW)** | | | **BB(Q1-HR)** | | | **BA(Q1-HR)** | | | **BC(Q1-HR)** | | | **CC(Q2-ARW)** | | | **CA(Q2-ARW)** | | | **CB(Q2-ARW)** | | |
| --- | --- | --- | --- | --- | --- | --- | --- | --- | --- | --- | --- | --- | --- | --- | --- | --- | --- | --- | --- | --- | --- | --- | --- | --- | --- | --- | --- |
| **Ala** | 18.19 | ± | 2.49 | 18.39 | ± | 1.81 | 16.96 | ± | 3.18 | 27.96 | ± | 2.21 | 25.42 | ± | 1.53 | 25.05 | ± | 0.97 | 24.19 | ± | 1.52 | 26.77 | ± | 1.49 | 26.23 | ± | 1.01 |
| **Arg** | 6.42 | ± | 0.78 | 7.04 | ± | 0.56 | 5.79 | ± | 0.60 | 6.98 | ± | 0.28 | 6.62 | ± | 0.50 | 6.59 | ± | 0.58 | 5.30 | ± | 0.53 | 5.78 | ± | 0.39 | 6.65 | ± | 0.27 |
| **Asn** | 2.01 | ± | 0.28 | 1.84 | ± | 0.24 | 2.53 | ± | 0.62 | 1.05 | ± | 0.17 | 1.20 | ± | 0.17 | 1.33 | ± | 0.08 | 1.42 | ± | 0.11 | 1.34 | ± | 0.11 | 1.06 | ± | 0.08 |
| **Asp** | 0.86 | ± | 0.14 | 0.67 | ± | 0.15 | 0.87 | ± | 0.25 | 0.60 | ± | 0.15 | 0.56 | ± | 0.16 | 0.32 | ± | 0.07 | 0.35 | ± | 0.06 | 0.42 | ± | 0.06 | 0.42 | ± | 0.11 |
| **Gln** | 26.76 | ± | 4.89 | 22.96 | ± | 3.76 | 30.62 | ± | 4.63 | 10.00 | ± | 1.93 | 14.48 | ± | 2.18 | 19.89 | ± | 3.22 | 22.02 | ± | 4.04 | 17.59 | ± | 2.57 | 17.41 | ± | 3.35 |
| **Glu** | 5.21 | ± | 0.36 | 6.26 | ± | 0.54 | 5.69 | ± | 0.26 | 5.81 | ± | 0.68 | 6.64 | ± | 0.86 | 5.88 | ± | 0.32 | 3.62 | ± | 0.24 | 3.69 | ± | 0.51 | 4.01 | ± | 0.57 |
| **Gly** | 4.19 | ± | 0.58 | 4.46 | ± | 0.47 | 3.77 | ± | 0.55 | 6.18 | ± | 0.23 | 5.78 | ± | 0.40 | 5.67 | ± | 0.29 | 4.75 | ± | 0.24 | 5.58 | ± | 0.33 | 5.45 | ± | 0.44 |
| **His** | 3.80 | ± | 0.37 | 3.47 | ± | 0.42 | 3.14 | ± | 0.26 | 2.97 | ± | 0.30 | 3.33 | ± | 0.30 | 3.12 | ± | 0.42 | 2.96 | ± | 0.21 | 2.96 | ± | 0.46 | 3.30 | ± | 0.28 |
| **Ile** | 1.57 | ± | 0.29 | 1.62 | ± | 0.15 | 1.43 | ± | 0.14 | 1.88 | ± | 0.17 | 1.62 | ± | 0.14 | 1.40 | ± | 0.17 | 1.58 | ± | 0.16 | 1.64 | ± | 0.14 | 1.54 | ± | 0.12 |
| **Leu** | 1.59 | ± | 0.38 | 1.88 | ± | 0.24 | 1.43 | ± | 0.38 | 2.67 | ± | 0.24 | 2.08 | ± | 0.21 | 1.90 | ± | 0.27 | 2.29 | ± | 0.22 | 2.31 | ± | 0.21 | 2.32 | ± | 0.17 |
| **Lys** | 5.91 | ± | 1.70 | 6.83 | ± | 1.19 | 4.44 | ± | 1.00 | 6.00 | ± | 1.03 | 5.33 | ± | 0.92 | 4.48 | ± | 0.72 | 5.27 | ± | 1.02 | 5.12 | ± | 0.95 | 4.77 | ± | 0.65 |
| **Met** | 0.75 | ± | 0.17 | 0.88 | ± | 0.11 | 0.59 | ± | 0.13 | 1.18 | ± | 0.10 | 1.04 | ± | 0.12 | 0.83 | ± | 0.10 | 1.06 | ± | 0.10 | 1.08 | ± | 0.13 | 1.10 | ± | 0.09 |
| **Phe** | 1.17 | ± | 0.16 | 1.30 | ± | 0.16 | 1.19 | ± | 0.18 | 1.89 | ± | 0.14 | 1.47 | ± | 0.10 | 1.52 | ± | 0.12 | 1.96 | ± | 0.35 | 2.10 | ± | 0.33 | 1.66 | ± | 0.12 |
| **Pro** | 6.64 | ± | 1.16 | 7.50 | ± | 0.44 | 7.74 | ± | 1.03 | 8.57 | ± | 1.41 | 9.93 | ± | 1.34 | 7.98 | ± | 1.03 | 10.05 | ± | 1.16 | 8.52 | ± | 1.18 | 9.83 | ± | 1.20 |
| **Ser** | 4.29 | ± | 0.46 | 4.13 | ± | 0.40 | 3.14 | ± | 0.43 | 5.16 | ± | 0.40 | 4.69 | ± | 0.59 | 4.34 | ± | 0.34 | 3.85 | ± | 0.20 | 4.54 | ± | 0.20 | 4.60 | ± | 0.49 |
| **Thr** | 2.28 | ± | 0.53 | 2.77 | ± | 0.44 | 2.88 | ± | 0.65 | 3.52 | ± | 0.56 | 2.68 | ± | 0.37 | 3.04 | ± | 0.38 | 3.22 | ± | 0.75 | 3.54 | ± | 0.87 | 3.30 | ± | 1.23 |
| **Trp** | 1.31 | ± | 0.27 | 1.23 | ± | 0.13 | 0.79 | ± | 0.07 | 1.07 | ± | 0.09 | 0.95 | ± | 0.06 | 1.00 | ± | 0.12 | 1.06 | ± | 0.15 | 1.16 | ± | 0.12 | 0.94 | ± | 0.03 |
| **Tyr** | 3.58 | ± | 0.57 | 3.26 | ± | 0.47 | 3.79 | ± | 0.73 | 2.91 | ± | 0.11 | 2.73 | ± | 0.17 | 2.96 | ± | 0.23 | 2.29 | ± | 0.12 | 2.77 | ± | 0.21 | 2.40 | ± | 0.17 |
| **Val** | 3.50 | ± | 0.38 | 3.55 | ± | 0.29 | 3.23 | ± | 0.31 | 3.61 | ± | 0.19 | 3.44 | ± | 0.26 | 2.72 | ± | 0.26 | 2.77 | ± | 0.15 | 3.10 | ± | 0.22 | 3.00 | ± | 0.22 |
| Ala: Alanine; Arg: Arginine; Asn: Asparagine; Asp: Aspartate; Gln: Glutamine; Glu: Glutamate; Gly: Glycine; His: Histidine; Ile: Isoleucine; Leu: Leucine; Lys: Lysine; Met: Methionine; Phe: Phenylalanine; Pro: Proline; Ser: Serine; Thr: Threonine; Trp: Tryptophan; Tyr: Tyrosine; Val: Valine. All individuals are F1 hybrid females obtained from the crosses of laboratory lineages: AA(Q1-HW), BB(Q1-HR), or CC(Q2-ARW). F1 females key: Nuclear genotype(Cytotype); Nuclear genotype: letters represent the maternally (first) and the paternally (second) inherited nuclear genotype; Cytotype: Mitochondrial group+S-symbionts; S-symbionts: A: *Arsenophonus*, H: *Hamiltonella*, R: *Rickettsia*, W: *Wolbachia.* | | | | | | | | | | | | | | | | | | | | | | | | | | | |

**Table S4**. Free amino acid profile in *B. tabaci* females on lantana, determined through HPLC analysis on individual females (n=8) (mean mol% ± SE)
