# Supplementary material for: Cytotype Affects the Capability of the Whitefly Bemisia tabaci MED Species To Feed and Oviposit on an Unfavorable Host Plant
Source: mBio. 2021 Nov 16;12(6):e00730-21. doi: 10.1128/mBio.00730-21 (PMC8593682; doi:10.1128/mBio.00730-21)
Supplement: TABLE S5 [file mbio.00730-21-st005.docx]

**Table S5**. Two-way ANOVA table investigating the effect of insect’s cytotype and genotype on the free amino acid content in *B. tabaci* females on lantana.

| **Amino Acid** | **Parameter** | **Df** | **Sum Sq** | **Mean Sq** | **F value** | **Pr(>F)** |  |
| --- | --- | --- | --- | --- | --- | --- | --- |
| Asp | Cytotype | 2 | 2.108 | 1.054 | 6.714 | 0.002 | ** |
|  | Cytotype:Genotype | 6 | 0.596 | 0.099 | 0.633 | 0.703 |  |
| Glu | Cytotype | 2 | 75.014 | 37.507 | 17.365 | <0.001 | *** |
|  | Cytotype:Genotype | 6 | 8.497 | 1.416 | 0.656 | 0.685 |  |
| Asn | Cytotype | 2 | 12.851 | 6.426 | 11.906 | <0.001 | *** |
|  | Cytotype:Genotype | 6 | 2.982 | 0.497 | 0.921 | 0.486 |  |
| Ser | Cytotype | 2 | 9.214 | 4.607 | 3.446 | 0.038 | * |
|  | Cytotype:Genotype | 6 | 11.762 | 1.960 | 1.466 | 0.204 |  |
| Gln | Cytotype | 2 | 1774.246 | 887.123 | 8.876 | <0.001 | *** |
|  | Cytotype:Genotype | 6 | 736.487 | 122.748 | 1.228 | 0.304 |  |
| His | Cytotype | 2 | 2.151 | 1.075 | 1.140 | 0.326 |  |
|  | Cytotype:Genotype | 6 | 2.883 | 0.480 | 0.509 | 0.799 |  |
| Gly | Cytotype | 2 | 37.353 | 18.677 | 13.917 | <0.001 | *** |
|  | Cytotype:Genotype | 6 | 6.305 | 1.051 | 0.783 | 0.586 |  |
| Thr | Cytotype | 2 | 6.226 | 3.113 | 0.807 | 0.451 |  |
|  | Cytotype:Genotype | 6 | 4.915 | 0.819 | 0.212 | 0.972 |  |
| Arg | Cytotype | 2 | 8.240 | 4.120 | 1.899 | 0.158 |  |
|  | Cytotype:Genotype | 6 | 14.414 | 2.402 | 1.107 | 0.368 |  |
| Ala | Cytotype | 2 | 1049.734 | 524.867 | 17.772 | <0.001 | *** |
|  | Cytotype:Genotype | 6 | 79.495 | 13.249 | 0.449 | 0.843 |  |
| Tyr | Cytotype | 2 | 13.731 | 6.865 | 6.132 | 0.004 | ** |
|  | Cytotype:Genotype | 6 | 2.379 | 0.396 | 0.354 | 0.905 |  |
| Val | Cytotype | 2 | 2.727 | 1.364 | 2.515 | 0.089 |  |
|  | Cytotype:Genotype | 6 | 4.508 | 0.751 | 1.385 | 0.234 |  |
| Met | Cytotype | 2 | 1.569 | 0.784 | 6.922 | 0.002 | ** |
|  | Cytotype:Genotype | 6 | 0.855 | 0.142 | 1.257 | 0.290 |  |
| Trp | Cytotype | 2 | 0.131 | 0.065 | 0.455 | 0.637 |  |
|  | Cytotype:Genotype | 6 | 1.475 | 0.246 | 1.710 | 0.133 |  |
| Phe | Cytotype | 2 | 5.704 | 2.852 | 8.694 | <0.001 | *** |
|  | Cytotype:Genotype | 6 | 1.717 | 0.286 | 0.872 | 0.520 |  |
| Ile | Cytotype | 2 | 0.101 | 0.051 | 0.217 | 0.806 |  |
|  | Cytotype:Genotype | 6 | 1.128 | 0.188 | 0.804 | 0.571 |  |
| Leu | Cytotype | 2 | 6.375 | 3.188 | 5.604 | 0.006 | ** |
|  | Cytotype:Genotype | 6 | 3.429 | 0.571 | 1.005 | 0.430 |  |
| Lys | Cytotype | 2 | 5.590 | 2.795 | 0.311 | 0.734 |  |
|  | Cytotype:Genotype | 6 | 33.557 | 5.593 | 0.623 | 0.711 |  |
| Pro | Cytotype | 2 | 59.907 | 29.954 | 2.899 | 0.062 |  |
|  | Cytotype:Genotype | 6 | 32.493 | 5.415 | 0.524 | 0.788 |  |
| Alanine; Arg: Arginine; Asn: Asparagine; Asp: Aspartate; Gln: Glutamine; Glu: Glutamate; Gly: Glycine; His: Histidine; Ile: Isoleucine; Leu: Leucine; Lys: Lysine; Met: Methionine; Phe: Phenylalanine; Pro: Proline; Ser: Serine; Thr: Threonine; Trp: Tryptophan; Tyr: Tyrosine; Val: Valine. Significance key: P<0.001 '***', P<0.01 '**', P<0.05 '*'. | | | | | | | |

### 
