# Supplementary material for: Cytotype Affects the Capability of the Whitefly Bemisia tabaci MED Species To Feed and Oviposit on an Unfavorable Host Plant
Source: mBio. 2021 Nov 16;12(6):e00730-21. doi: 10.1128/mBio.00730-21 (PMC8593682; doi:10.1128/mBio.00730-21)
Supplement: TABLE S6 [file mbio.00730-21-st006.docx]

**Table S6**. P values from multiple comparisons (Tukey’s pairwise comparison) of the free amino acid content in *B. tabaci* females on lantana between cytotypes. For each amino acid, analyses were carried out only when the cytotype factor had a significant effect on the amino acid content in two-way ANOVA (**Fig. 6**, **Table S5**).

| Amino acid | Contrast | p-value | |
| --- | --- | --- | --- |
| Asp | Q1-HW == Q1-HR | 0.026 | * |
|  | Q1-HW == Q2-ARW | 0.002 | ** |
|  | Q1-HR == Q2-ARW | 0.683 |  |
| Glu | Q1-HW == Q1-HR | 0.630 |  |
|  | Q1-HW == Q2-ARW | <0.001 | *** |
|  | Q1-HR == Q2-ARW | <0.001 | *** |
| Asn | Q1-HW == Q1-HR | <0.001 | *** |
|  | Q1-HW == Q2-ARW | <0.001 | *** |
|  | Q1-HR == Q2-ARW | 0.923 |  |
| Ser | Q1-HW == Q1-HR | 0.029 | * |
|  | Q1-HW == Q2-ARW | 0.335 |  |
|  | Q1-HR == Q2-ARW | 0.459 |  |
| Gln | Q1-HW == Q1-HR | <0.001 | *** |
|  | Q1-HW == Q2-ARW | 0.024 | * |
|  | Q1-HR == Q2-ARW | 0.316 |  |
| Gly | Q1-HW == Q1-HR | <0.001 | *** |
|  | Q1-HW == Q2-ARW | 0.004 | ** |
|  | Q1-HR == Q2-ARW | 0.161 |  |
| Ala | Q1-HW == Q1-HR | <0.001 | *** |
|  | Q1-HW == Q2-ARW | <0.001 | *** |
|  | Q1-HR == Q2-ARW | 0.963 |  |
| Tyr | Q1-HW == Q1-HR | 0.077 |  |
|  | Q1-HW == Q2-ARW | 0.003 | ** |
|  | Q1-HR == Q2-ARW | 0.432 |  |
| Met | Q1-HW == Q1-HR | 0.017 | * |
|  | Q1-HW == Q2-ARW | 0.002 | ** |
|  | Q1-HR == Q2-ARW | 0.785 |  |
| Phe | Q1-HW == Q1-HR | 0.041 | * |
|  | Q1-HW == Q2-ARW | <0.001 | *** |
|  | Q1-HR == Q2-ARW | 0.230 |  |
| Leu | Q1-HW == Q1-HR | 0.025 | * |
|  | Q1-HW == Q2-ARW | 0.009 | ** |
|  | Q1-HR == Q2-ARW | 0.917 |  |
| Cytotype key: Mitochondrial group+S-symbionts; S-symbionts : A: *Arsenophonus*, H: *Hamiltonella*, R: *Rickettsia*, W: *Wolbachia*. Significance key: P<0.001 '***', P<0.01 '**', P<0.05 '*' | | | |
