# Supplementary material for: Cytotype Affects the Capability of the Whitefly Bemisia tabaci MED Species To Feed and Oviposit on an Unfavorable Host Plant
Source: mBio. 2021 Nov 16;12(6):e00730-21. doi: 10.1128/mBio.00730-21 (PMC8593682; doi:10.1128/mBio.00730-21)
Supplement: TABLE S7 [file mbio.00730-21-st007.docx]

| **Plant** | **Stage** | **Leaves** |
| --- | --- | --- |
| *Hibiscus moscheutos* | 2-month-old seedlings, 13^th^ true leaf stage | 9^th^, 10^th^, 11^th^ true leaves |
| *Lantana camara* | 1-month-old branchings | 3^rd^, 4^th^, 5^th^ leaves from the branches apex |
| *Nicotiana tabacum* | 2-month-old seedlings, 8^th^ true leaf stage | 6^th^ true leaf |

**Table S7.** Plants parts used for the free amino acids analysis of plant leaves
